# Effect of *FABP4* Gene Polymorphisms on Fatty Acid Composition, Chemical Composition, and Carcass Traits in Sonid Sheep

**DOI:** 10.3390/ani15020226

**Published:** 2025-01-15

**Authors:** Jiada Xiang, Haofan Li, Zhaoxin Guo, Terigele Li, Takahisa Yamada, Xihe Li, Siqin Bao, Lai Da, Gerelt Borjigin, Ming Cang, Bin Tong

**Affiliations:** 1 The State Key Laboratory of Reproductive Regulation and Breeding of Grassland Livestock, School of Life Sciences, Inner Mongolia University, Hohhot 010021, China; 2 Inner Mongolia Agriculture Animal Husbandry Fishery and Biology Experiment Research Centre, Inner Mongolia Agricultural University, Hohhot 010010, China; 3 Department of Agrobiology, Faculty of Agriculture, Niigata University, Niigata 950-2181, Japan; 4 Institute of Animal Science, Inner Mongolia Academy of Agricultural and Animal Husbandry Sciences, Hohhot 010031, China; 5 College of Food Science and Engineering, Inner Mongolia Agricultural University, Hohhot 010010, China

**Keywords:** association, *FABP4* gene, fatty acids, genetic variants, Sonid sheep

## Abstract

Fatty acids (FAs) are a group of organic compounds that are regulated by polygenic and environmental factors and affect the taste, nutritional value, and quality of meat. Lamb meat is rich in FAs required by the human body, which has directed more attention to sheep research and meat production. The fatty acid-binding protein 4 (*FABP4*) gene is considered a candidate gene that can affect FA composition in livestock. Therefore, the aim of this study was to screen for genetic polymorphisms of *FABP4* and confirm the association between these polymorphisms and FAs, chemical composition, and carcass traits in Sonid lambs. The results of the association study showed that g.57764667T>C, g.57764436T>G, g.57764242G>A, and g.57757988A>G were associated with the composition of certain long-chain FAs, and g.57764242G>A, g.57764436T>G, and g.57758026G>A were associated with free amino acid levels. In addition, g.57764667T>C and g.57757988A>G were associated with carcass weight and live weight in Sonid lambs. Therefore, the polymorphisms of the *FABP4* gene are expected to be a genetic selection marker for superior traits in Sonid sheep breeding, which also provides new insights into how the ovine *FABP4* gene affects traits of lamb quality.

## Simple Summary

This study focused on the association between the diversity of *FABP4* loci and fatty acid (FA) composition, chemical composition, and carcass traits in the *longissimus thoracis* (LT) of Sonid sheep. A sequence analysis identified nine novel polymorphisms in the *FABP4* gene of Sonid sheep. Among them, the g.57764667T>C, g.57764436T>G, g.57764242G>A, and g.57757988A>G mutations are associated with the composition of some long-chain FAs, including ω-3 and ω-6, and g.5776442G>A is associated with crude protein, crude fat, and free amino acid levels, while g.57764436T>G and g.57758026G>A are associated with free amino acid levels. In addition, the g.57764667T>C and g.57775988A>G mutations are associated with carcass weight and live weight. Therefore, these polymorphisms are expected to serve as genetic selection markers for meat quality and carcass traits in Sonid sheep.

## 1. Introduction

With its unique taste and high nutritional value, lamb is widely preferred by the general public. China’s demand for lamb meat is gradually increasing, and the production and scale of lamb meat are also expanding [1]. According to related reports, the total production of lamb in China was 5.3126 million tons in 2023, an increase of 1.3% from the previous year [2]. With the continuous rise in people’s living standards in China, the demand for high-quality lamb meat is also gradually increasing [3]. Sonid sheep is a typical homologous breed of Mongolia sheep. They are fat-tailed and produce quality meat and carpet wool. Sonid sheep have fine body conformation, strong walking ability, and admirable adaptation to grassland and desert areas in northern China and southern Mongolia. There are approximately 2.206 million Sonid sheep in the Inner Mongolia Autonomous Region [4]. In view of Sonid sheep’s tender and juicy meat, firm muscle layer, high nutritional value, and light odor, more and more consumers have a demand for it [5,6].

Lamb meat is rich in fatty acids (FAs). Also, the proportions of saturated fatty acid (SFA), monounsaturated fatty acid (MUFA), and polyunsaturated fatty acid (PUFA) in meat lamb are of great significance for maintaining normal physiological function in the human body [7,8]. The flavor of lamb perceived by consumers is generally closely related to the content of certain beneficial FAs [9]. Amino acid content is also an important substance affecting lamb meat quality. Relevant studies have shown that adding 1% yeast culture to the diet can affect the content of key amino acids such as Lysine (Lys) and Arginine (Arg) in sheep muscle, leading to changes in lamb meat quality, especially lamb meat flavor [10]. Carcass traits are also one of the important indicators for sheep breeding. A higher dressing percentage means a higher efficiency and yield of meat production, which can indirectly increase the income of herdsmen [11]. FAs, amino acid content, and carcass traits are mainly restricted by environmental and genetic factors [12,13,14,15,16]. In order to further optimize the texture and flavor of Sonid mutton, expand its market, and improve economic benefits for herdsmen, it is very important to explore the genetic mechanisms of FAs, amino acid composition, and carcass traits in sheep and to apply molecular mark-assisted selection (MAS) in the breeding process [17,18,19,20]. However, compared to other major domestic animals such as pigs [21,22,23] and cattle [24,25], the discovery and exploration of candidate genes affecting FAs, chemical composition, and carcass traits in sheep are still limited, and research on candidate genes in Sonid sheep living in desert or semi-desert Sonid grasslands is even rarer. Therefore, it is necessary to screen and study candidate genes for Sonid sheep breeding.

Fatty acid-binding protein 4 (FABP4) is a member of the fatty acid-binding protein (FABP) family. The β-barrel structure of the FABP4 protein core can bind to long-chain FAs in a non-covalent form, so it has a high affinity for FAs [26,27]. By recognizing and binding to FAs on the cell membrane, FABP4 transfers extracellular FAs to the cytoplasm and further enters mitochondria and other organelles to participate in biochemical reactions such as FA oxidation, thereby reducing the “adverse reactions” caused by the accumulation of FAs in cells [27,28]. The *FABP4* gene is located on chromosome 9 of the ovine genome, is 5871 bp in length, covering four exons, and is present in many organs and tissues [29,30,31]. Polymorphisms in *FABP4* could influence FAs, fat content, and carcass traits in mammals. Related studies have indicated that in bovine intramuscular preadipocytes, an increase in lipid droplets after the addition of palmitoleic acid may be one of the reasons for the difference in palmitoleic acid content between *FABP4* polymorphisms with and without the addition [32]. Five single-nucleotide polymorphism (SNP) loci with significant effects on fat content were found in the *FABP4* gene of Yanbian cattle, which provided candidate markers for fat metabolism and the breeding of cattle [33]. The c.220A>G polymorphism of the *FABP4* gene was significantly associated with carcass traits and body size in Chinese Qinchuan cattle, while the g.3473A>T polymorphism was significantly associated with hot carcass weight in Korean cattle [34,35]. Furthermore, variation in porcine *FABP4* has also been reported to be associated with IMF content in pork and possibly with growth in Duroc pigs [36]. Moreover, a study has shown the effect of haplotypes in the intron of the *FABP4* gene on sheep growth and carcass traits [37]. Thus, the *FABP4* gene is of great value as a potential candidate gene for sheep meat. However, there is still a lack of information on the relationship of *FABP4* polymorphism with FAs, chemical composition, and carcass traits in sheep.

Therefore, this study focused on two objectives: to identify novel *FABP4* mutations in Sonid sheep and to analyze the association of these mutations with the FA types, meat quality, and carcass traits of Sonid sheep. This study provides a new effective marker for Sonid sheep breeding and a new perspective for exploring the effect of the *FABP4* gene on meat quality traits in sheep.

## 2. Materials and Methods

### 2.1. Statement of Ethics

Animal welfare and experimental procedures were carried out in compliance with the guidelines for the Administration of Affairs Concerning Experimental Animals set forth by the Ministry of Science and Technology, China, in 2004. The research protocol received approval from the Institutional Animal Care and Use Ethics Committee at Inner Mongolia University on 15 May 2015, under the authorization number IMU-2015-03 for the execution of animal studies.

### 2.2. Lamb Sample Preparation

A total of 276 six-month-old (surgical castration was performed within one month after birth) male lambs born in 2023 were randomly selected for this study, and all the experimental animals were healthy with free access to food and water and natural lighting. During the period after birth and before weaning (at approximately 3 months old), these lambs fed on the milk of ewes. Post-weaning, they were arranged to graze on the same Sonid grassland; the Sonid grassland is a desert steppe and semi-desert steppe, and most of the vegetation in the grassland is plants of the herb family and liliaceae family, including stipa breviflora, cryptotia longifolia, artemisia longifolia, and other plants. These plants are the food of Sonid lambs and contribute to the growth of Sonid lambs [38]. When the lambs were six months old, they were sent to the slaughterhouse in Sonid Left Banner for slaughter. The 276 lambs were born to different ewes, whose parents were from a group of more than 20 unrelated rams, showing that these lambs had a wide enough genetic background and no specific parents.

The slaughtering of the lambs adhered to the Chinese industry standard (NY/T 1564-2021) [39]. After slaughter, approximately 30 g of *longissimus thoracis* (LT) muscle tissues (between the 12th and 13th ribs) from the left half of the carcasses of the 276 lambs was excised immediately after 20 min post-exsanguination, and snap-frozen in liquid nitrogen after the fat and connective tissue were trimmed, and each muscle tissue was collected into 4–5 cryopreserved tubes and stored at −80 °C until FA analysis. Of the 276 lambs, 10 lambs were randomly chosen for a tissue expression profile analysis of the *FABP4* gene (including in the heart, liver, spleen, lung, subcutaneous fat, kidney, large intestine, small intestine, *semitendinosus*, aponeurosis, LT, and stomach). Additionally, of the samples taken from the 276 lambs, 50 g LT samples removed (between the 12th and 13th ribs) from the right half of the carcasses of 111 lambs were randomly selected for chemical composition determination (including crude fat, crude protein, and free amino acid), and carcass traits (including at 45 min (pH_1_) and 24 h (pH_2_) and color determination) were measured in the area between the 12th and 13th ribs. Meanwhile, the dressing percentage of these 111 lambs was also calculated.

### 2.3. Real-Time Quantitative PCR (RT-qPCR)

Total RNA of the LT muscle tissue of Sonid sheep was isolated utilizing the RNAiso plus kit (Takara, Dalian, China) and reverse-transcribed to cDNA utilizing the primeScript^™^ RT Reagent Kit with gDNA Eraser (Takara, Dalian, China). RT-qPCR analysis was performed with an SYBR Green master mix and the primers listed in Table 1, employing a system from Bio-Rad (Bio-Rad, Hercules, CA, USA). The *GAPDH* gene served as an internal housekeeping gene for normalization in this experiment [40]. The relative expression levels of the *FABP4* gene were calculated using the 2^−∆∆Ct^ method [41].

### 2.4. Resequencing and Variant Detection in FABP4

DNA from 20 Sonid lambs was analyzed to identify variations within the *FABP4* gene. The design of specific PCR primers for sheep was facilitated by primer 5.0 software (Premier Biosoft International, Palo Alto, CA, USA) (Table S1). The targeted genomic regions encompassed 2000 bp upstream of the promoter, all exons, all introns, and 500 bp of the 3′ untranslated regions (3′ UTRs) of the ovine *FABP4* gene (NCBI reference sequence: NC_056062.1) (Table S1). Table S1 provides a detailed description of the PCR amplification process, including the annealing temperatures and target fragment length for each reaction. The PCR products underwent analysis using 1.0% agarose gel electrophoresis to assess quality and quantity. Sangon Biotech (Shanghai, China) performed the sequencing of the products.

### 2.5. Polymorphism Genotyping Using iPLEX MassARRAY

The MassARRAY^®^ SNP genotyping system (Biosciences, San Diego, CA, USA) was utilized to examine the genotypes of nine identified polymorphisms in the *FABP4* gene of a cohort of 276 Sonid sheep. The PCR and extension primers of *FABP4* were designed from sequences containing each target mutation and ~100 upstream and downstream bases via the Assay Design Suite v3.0. These primers were designed based on sequences that encompassed the specific mutations and extended roughly 100 bases both upstream and downstream (Table S2). The Sequenom MassARRAY iPLEX platform was the tool of choice for determining the genotypes of the individual alleles. An analysis of the gathered data was conducted using MassARRAY Typer 4.0 analyzer software (Biosciences, San Diego, CA, USA) [42].

### 2.6. Determination of Fatty Acid Composition

The intramuscular lipids were extracted from LT samples to produce FA methyl ester (FAME) by a modified Folch method [43]. The extracted samples (200 mg) were methylated separately using base (0.5 mol/L CH_3_ONa) and acid (14% BF_3_CH_3_OH) reagents, and each process took place in a 40 °C water bath for 20 min [44]. After cooling, three mL of n-hexane and the layer were removed by oscillating extraction, and then FAME was obtained.

The FAME analysis followed the recommendations made by Dugan and Wood, 2018 [45]. The FAMEs were quantified by gas chromatography (GC) (Clarus 690 GC, PerkinElmer, Waltham, MA, USA). The running conditions were as follows: The capillary column had a size of 100 m × 0.2 μm × 0.25 mm (SP-2560, Sigma-Aldrich, Shanghai, China). The initial temperature of the injection port and detector was set to 250 °C. The column temperature was held steady at 80 °C for 3 min before being increased to 250 °C at a rate of 10 °C/min and kept for 15 min. Helium was supplied at a flow rate of 1 mL/min, with a splitting ratio set at 20:1. The FAMEs were quantified using the chromatographic peak area and C11:0 internal standard (LGC Labor GmbH, Augsburg, Germany)-based calculations. The specific FA peaks were identified by the GC retention times of known standards (Supelco 37 Component FAME Mix, Sigma-Aldrich, Shanghai, China) and conjugated linoleic acid (CLA, Shanghai ANPEL Scientific Instrument, Shanghai, China). Three repetitions were performed for all measurements, and the unit for FA is mg/100 g.

### 2.7. Measurements of Carcass Traits

The slaughtering procedure of experimental sheep was conducted in accordance with the Chinese agricultural industry standard NY/T 3469-2019 [46]. The dressing percentage was calculated based on the percentage of the individual hot carcass weight measure to the live weight measure. Live weight was measured on an empty stomach at 12 h before slaughter, and hot carcass weight was measured approximately 10 min post-exsanguination. The pH values at pH_1_ and pH_2_ were measured using pH-STAR (Matthaus, Bavaria, Germany). The pH meter was calibrated with standard buffers at pH = 4.64 and pH 7.00, at 25 °C, and the temperature compensation was set during the calibration process. The pH probe was inserted directly into the LT sample, and at the same time, three consecutive measurements were taken at different locations of each sample to obtain an average value. The lamb meat color was determined by the CIE L*, a*, b* (L*—lightness; a*—redness; and b*—yellowness) system using a reflective colorimeter (WSC-S, Shanghai Precision Science Instrument, China) with illuminant D65 as the light source, an 8° observer angle, and an 8 mm measuring aperture.

### 2.8. Measurements of Chemical Composition

For crude fat measurement, the petroleum ether extract was obtained through Soxhlet fat extraction using extractor B-811 (Buchi, Flavell, Switzerland) [47]. The crude protein in the LT muscle was determined using the Kjeldahl method described in the China National Food Safety Standard (GB 5009.5–2016) [48]. The K-360 automatic Kjeldahl nitrogen analyzer (Buchi, Flavell, Switzerland) was utilized to streamline the processes of liquid addition, distillation, titration, and data recording for the calculation of protein content (Kjeldahl N x 6.25). For free amino acids, the 0.1 g sample was weighed and mixed with 0.5 mL PBS and then centrifuged at 4 °C at 2500 r/min for 5 min. The same volume of supernatant was collected and thoroughly mixed with 10% trichloroacetic acid, stored in an ice bath for 30 min [49], and filtered through a water-soluble filter with a pore size of 0.22 μm. The free amino acids were quantified using the L-8900 high-speed amino acid analyzer (Hitachi, Tokyo, Japan) equipped with a cation exchange column. A ninhydrin solution served as the post-column derivative reagent. The N-derived amino acids were detected at the wavelengths of 570 nm and 440 nm using a sample size of 50 μL, a column temperature set to 130 °C, and a flow rate maintained at 0.25 mL/min [50].

### 2.9. Bioinformatics Analysis

The alignment of multiple sequences and the creation of molecular phylogenetic trees were conducted using NCBI Blast (https://blast.ncbi.nlm.nih.gov/Blast.cgi, accessed on 23 January 2024) and MEGA-X. The transcription factor binding sites in the *FABP4* promoter were identified with JASPAR (http://jaspar.genereg.net/, accessed on 24 January 2024) using standard settings for the highest matrix similarity [51].

### 2.10. Statistical Analysis

Calculations of population genetic metrics, including the polymorphism information content (PIC), numbers of effective alleles (n_e_), observed heterozygosity (Ho), and expected heterozygosity (He), were performed using the methods developed by Nei and Roychoudhury (1974). The chi-squared test of independence was used to calculate the allele frequencies of each mutation. Linkage disequilibrium (LD) analysis was conducted using HaploView 4.2 software, considering D’ and *r*^2^ values [52]. The relationship between FA composition/class and genotypes of the five variants was examined using SPSS 27.0 software (SPSS, Chicago, IL, USA), as reported by Guo et al. [53]. A statistical linear model—Y_i_ = μ + G_i_ + e_i_—was applied, where Y_i_ represents the observed value of each trait, μ is the average value of each measurement, G_i_ denotes the fixed effect of genotype, and e_i_ stands for the standard error. The *p*-value was adjusted using Bonferroni correction [35].

## 3. Results

### 3.1. Fatty Acid Profiles of Longissimus Thoracis Muscle Tissue

**Figure 1:**
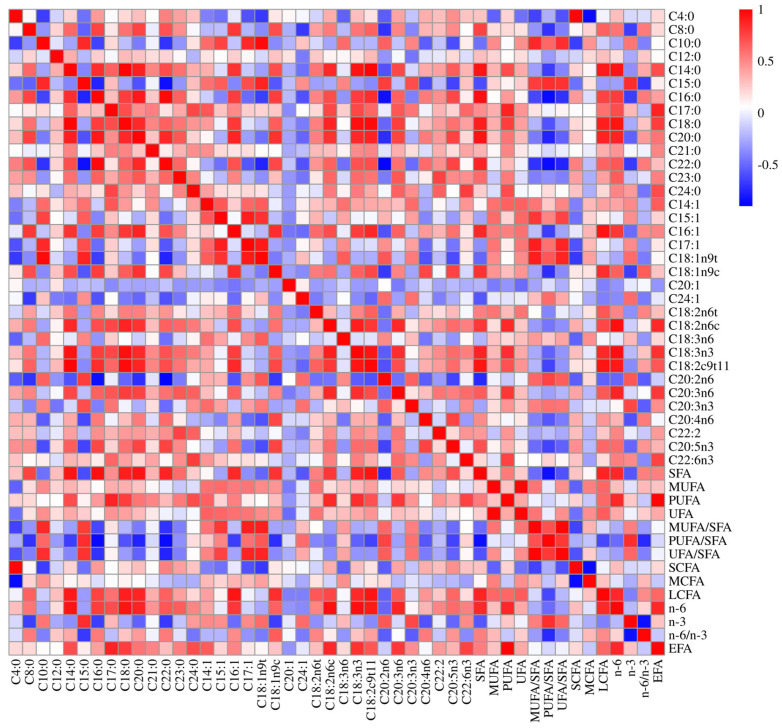
A correlation plot for the correlation analysis between FA composition and class trait pairs. Red shading indicates a positive correlation, while blue shading indicates a negative correlation. The intensity of the color is proportional to the magnitude of the correlation coefficient.

The FA composition and classes in the Sonid sheep LT muscle were analyzed, and the relevant summary can be viewed in Table S3. The table lists 14 types of SFAs, 8 types of MUFAs, and 12 types of PUFAs present in the LT muscle. Graphical representations of the correlation analyses between any two traits of the FA components/classes are shown in Figure 1, and the correlation coefficients and their respective *p*-values are given in Table S4.

The content of C18:2n6t in the LT muscle was negatively correlated with C20:2n6 (*r* = −0.367, *p* < 0.01) and n-3 (*r* = −0.146, *p* < 0.05), and it was positively correlated with C18:3n3, C18:2c9t11, MUFA, unsaturated fatty acid (UFA), MUFA/SFA, long-chain fatty acid (LCFA), and n-6, with *r* values ranging from 0.263 to 0.343 and *p* < 0.01 (Figure 1, Table S4). The C18:2n6c content of the LT muscle was negatively correlated with PUFA/SFA (*r* = −0.200, *p* < 0.01), and it was significantly and positively correlated with C18:3n3, C20:3n3, PUFA, LCFA, n-6, and essential fatty acid (EFA), with *r* values ranging from 0.580 to 0.916 and *p* < 0.01 (Figure 1, Table S4). The C18:3n3 content of the LT muscle was negatively correlated with C20:2n6 (*r* = −0.240 and *p* < 0.01) and PUFA/SFA (*r* = −0.321 and *p* < 0.01), and it was significantly and positively correlated with C18:2c9t11, C20:3n6, LCFA, n-6, and EFA, with *r* values ranging from 0.501 to 0.808 and *p* < 0.01 (Figure 1, Table S4). The C18:2c9t11 content of the LT muscle was negatively correlated with C20:2n6 (*r* = −0.364 and *p* < 0.01) and PUFA/SFA (*r* = −0.471 and *p* < 0.01), and it was significantly and positively correlated with SFA, PUFA, LCFA, n-6, and EFA, with *r* values ranging from 0.597 to 0.845 and *p* < 0.01 (Figure 1, Table S4).

### 3.2. FABP4 Gene Expression Profile and Phylogenetic Tree

**Figure 2:**
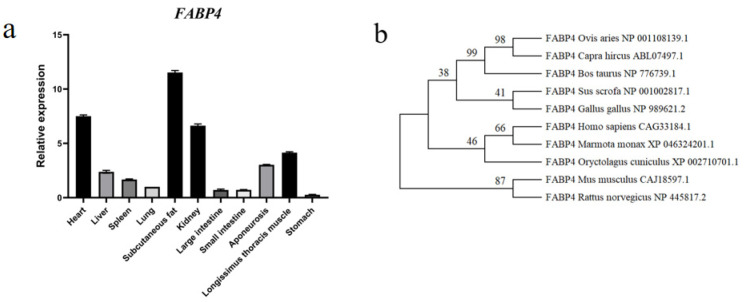
*FABP4* gene expression profiles and phylogenetic tree. (**a**) Relative expression levels of the *FABP4* gene in 11 tissues of Sonid sheep. Data are presented as means ± standard errors. (**b**) Phylogenetic tree based on the homologous amino acid sequence of FABP4.

The gene expression profile of the *FABP4* gene in 11 tissues of Sonid lambs is depicted in Figure 2a. *FABP4* was highly expressed in the heart, subcutaneous fat, kidney, and LT (Figure 2a). To build a phylogenetic tree for the gene analogous to ovine *FABP4*, the amino acid sequences of FABP4 from model organisms and domesticated animals were selected. Subsequently, the maximum parsimony method was employed to construct a molecular phylogenetic tree, utilizing 1000 Bootstrap replications within MEGA 11.0.13 software. The results showed 98% homology between *Ovis aries* and *Capra hircus*, and a 99% homology of *Bos taurus* with *Ovis aries* and *Capra hircus* (Figure 2b).

### 3.3. Variant Identification in Sonid Sheep FABP4 Gene

**Figure 3:**
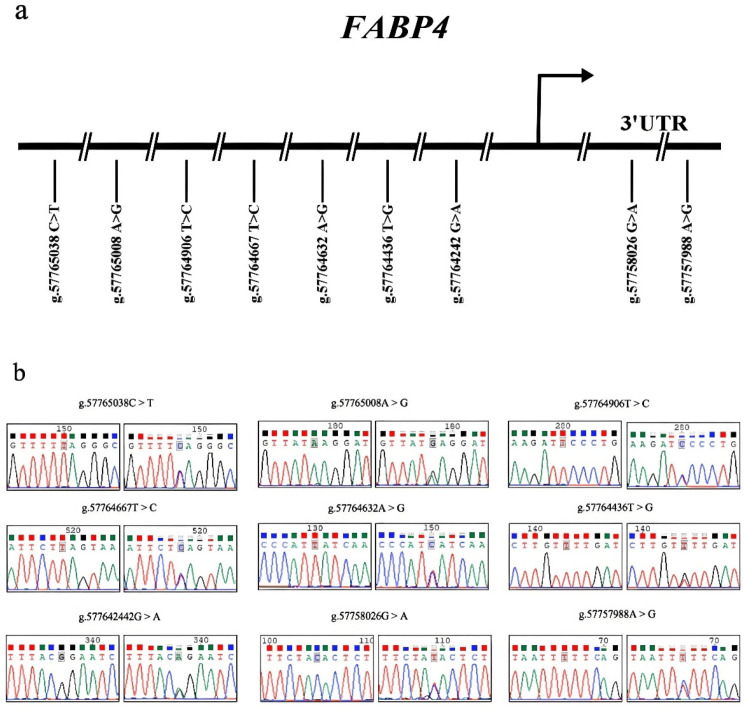
The physical locations of the nine novel variants in the Sonid sheep *FABP4* gene. (**a**) The physical locations of the nine novel variants in the Sonid sheep *FABP4* gene. (**b**) Nucleotide substitutions of the nine *FABP4* variants are shown. The variant sites are located on chromosome 9 as per the NCBI reference sequence: NC_056062.1.

The directed sequence identified nine novel variants in the *FABP4* gene of Sonid sheep. Among them, seven mutations (g.57765038C>T (rs421288924), g.57765008A>G (rs399102867), g.57764906T>C (rs409321853), g.57764667T>C (rs430602792), g.57764632A>G (rs406525689), g.57764436T>G (rs416595991), and g.57764242G>A (rs416009905)) are in the promoter regions of *FABP4*, respectively (Figure 3a,b), and two mutations (g.57758026G>A (rs430172432) and g.57757988A>G (rs414987233)) are in the 3′ untranslated region (3′ UTR) of *FABP4*, respectively (Figure 3a,b).

**Figure 4:**
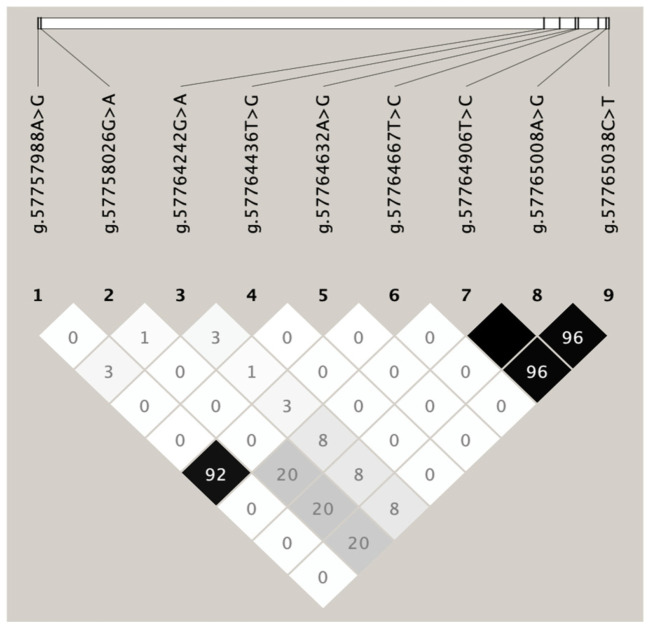
Linkage disequilibrium estimated among *FABP4* variations in the Sonid sheep population. The numbers in the figure represent the values of *r*^2^ × 100.

For the nine mutations identified in this study, the genotypical and allelic frequencies in the Sonid population, along with the genetic indices, are listed in Table S5. No significant departures at the 5% level were detected by any test for each variant in Sonid sheep (Table S5). This study conducted a linkage analysis of D’ and *r*^2^ mutations in Sonid sheep and found that the g.57764906T>C mutation site and the g.57765008A>G mutation site had *r*^2^ = 1.000, indicating that the g.57764906T>C and g.57765008A>G mutation sites were completely in LD in Sonid sheep (Figure 4). Thus, these LD groups were analyzed together and marked as a single locus, named LD1. The values of D’ and *r*^2^ are shown in Table S6. Moreover, this LD is also present in other sheep breeds or populations (http://asia.ensembl.org/Ovis_aries_rambouillet/Variation/Explore?v=rs1090132668, accessed on 22 January 2024).

### 3.4. Association Analysis of Novel Variants in FABP4 with Fatty Acid

The results of significant association between novel variants of *FABP4* and FA compositions/classes in the LT of Sonid lambs are shown in Table 2, with full results provided in Table S7. At the g.57765038C>T site, the C16:1 and C18:1n9c contents of individuals with the TT genotype were significantly higher than those of individuals with the CT genotype (*p* < 0.05, Table 2). For the g.57765008A>G site of LD1, the C16:1 and C18:1n9c contents of individuals with the GG genotype were significantly higher than those of individuals with the AG genotype (*p* < 0.05, Table 2). At the g.57764667T>C site, the C22:6n3 content of individuals with the TC genotype was significantly higher than that of those with the TT genotype (*p* < 0.01, Table 2). For the g.57764436T>G site, the C18:2n6c content of individuals with the TG genotype was significantly higher than that of those with the TT genotype (*p* < 0.05, Table 2); compared to the TG genotype, the TT genotype had significantly higher C18:1n9t (*p* < 0.05) and C20:3n3 (*p* < 0.01) contents (Table 2). At the g.57764242G>A site, the C18:1n9c, C18:2n6c, and C18:3n3 contents of individuals with the GG genotype were significantly higher than those of individuals with the GA genotype (*p* < 0.05, Table 2). In addition, individuals with the AG genotype of the g.57757988A>G mutation had a significantly higher C22:6n3 content than those with the AA genotype in Sonid lambs (*p* < 0.01) (Table 2).

### 3.5. Association Analysis of Variants in FABP4 with Chemical Composition

The g.57764632A>G mutation was removed because its genotyping sample size was less than ten. There were not any associations between the g.57765038C>T, g.57765008A>G-LD1, g.57764667T>C, and g.57757988A>G mutations and chemical composition in Sonid sheep (Table S8). For the g.57764436T>G variation, the TG genotype had significantly higher free Aspartic acid (Asp) and free Lys levels than those of the TT genotype (*p* < 0.05) (Table 3). For the g.57764242G>A site, the AA genotype had significantly higher crude protein levels than the GG genotype (*p* < 0.05); compared to the GA genotype and AA genotype, the GG genotype had significantly higher crude fat levels (*p* < 0.05), and the GG genotype also had significantly higher Asp levels than the GA genotype (*p* < 0.05) (Table 3). In addition, animals with the GG genotype of g.57758026G>A had significantly higher free Alanine (Ala) levels when compared to those of the GG genotype *(p* < 0.05) in Sonid lambs (Table 3).

### 3.6. Association Analysis of Variants in FABP4 with Carcass Traits

The g.57764436T>G mutation was removed because its genotyping sample size was less than ten. There were not any associations between the g.57765038C>T, g.57765008A>G-LD1, and g.57758026G>A mutations and carcass traits in Sonid lambs (Table S9). For the g.57764667T>C mutation, the TT genotype had a significantly higher carcass weight (*p* < 0.05) and live weight (*p* < 0.01) when compared to those of the TC genotype (Table 4). For the g.57764242G>A variation, the GG genotype had a significantly higher carcass weight (*p* < 0.05) and live weight (*p* < 0.01) than the AA genotype (Table 4). Furthermore, individuals with the AA genotype of the g.57757988A>G variation had a significantly higher carcass weight (*p* < 0.05) and live weight (*p* < 0.01) than the AG genotype (Table 4). There was no relationship of the three variants in *FABP4* with meat color and pH in Sonid lambs (Table 4).

## 4. Discussion

FABP4, a member of the family of fatty acid-binding proteins, is a gene located on chromosome 9 in sheep, covering four exons, and is present in many organs and tissues [30,31,34]. FABP4 is an intracellular protein that has effects on intracellular lipid binding, metabolism, and signaling. It is regarded as the main protein expressed in adipocytes in non-covalent binding to FAs on the cell membrane, transporting FAs into the cells and then transporting them to the organelles involved in biochemical reactions such as FA oxidation [26,28,54,55]. The moderately variable SNPs of the *FABP4* gene can affect the meat quality traits of Yanbian cattle. Among them, the g.3691G>A (also known as rs110652478G>A) polymorphism was significantly associated with water, fat, and protein contents in Yanbian cattle. The six SNPs in the study all had significant effects on the fat content, protein content, and marbling grade of Yanbian cattle [33]. In addition, in the *FABP4* gene of Korean cattle, the g.3691G>A (rs110652478G>A) polymorphism is also significantly associated with marbling grade and meat quality grade [56]. These results indicate a relationship between the *FABP4* gene and fat content. However, FA-related studies of the *FABP4* gene are mainly concentrated in other domestic animals such as cattle or pig, and this topic is rarely investigated in sheep [57]. In this study, we aimed to address this gap by profiling *FABP4* gene polymorphisms in Sonid sheep, to investigate their effects on FAs, chemical composition, and carcass traits, and to explore the possible reasons for the differences.

A promoter is a cis-acting element located upstream of the start point of gene transcription which has the ability to regulate the initiation and intensity of gene transcription and is a key component in ensuring the normal expression of genes [58,59,60]. Some of the changes in physiological morphology may not be caused by mutations in the coding region, but by mutations in the promoter, which have significant effects on the body’s function [61,62]. It has been reported that supplementation of omega-3 in Tattykeel Australian White lambs increased the docosahexaenoic acid (DHA) content in the intramuscular fat due to the enhanced expression of the *FABP4* gene [63]. In this study, we found that for the g.57764667T>C SNP in the promoter of the *FABP4* gene, the DHA content of the heterozygous genotype was significantly higher than that of the homozygous genotype. In addition, Lys is one of the essential amino acids in mammals. It can regulate bone growth, immune regulation, and energy metabolism in animals [64]. Hence, we analyzed amino acids in the LT muscle of Sonid lambs and found that the g.57764436T>G and g.57764242G>A mutations in *FABP4* are associated with Lys and Asp in Sonid lambs, respectively. Meanwhile, we found that the g.57764667T>C and g.57764242G>A SNPs in the promoter of the *FABP4* gene are also related to the carcass traits of Sonid lambs. Therefore, *FABP4* could be considered a candidate gene for influencing meat quality, amino acid synthesis, and carcass traits in Sonid lambs. Previous studies have shown that point mutations in the promoter region can affect the binding of transcription factors, thereby affecting the expression of corresponding genes [65]. According to the results of the present study, the g.57764242G>A polymorphism in the *FABP4* gene promoter is associated with C18:1n9c content, Asp content, and live weight and carcass weight. Therefore, we speculate that the g.57764242G>A mutation of the *FABP4* gene promoter may reduce the binding of transcription factors to the promoter, further reducing the transcription and translation level of the *FABP4* gene, resulting in the decreased expression of the *FABP4* gene and further leading to changes in the FA content, amino acid content, and carcass weight of Sonid sheep. This affects the meat quality and carcass traits of Sonid lambs. However, further experiments are needed to confirm the exact mechanism. For example, transcription factor activity can be assessed by the luciferase reporter assay, electrophoretic mobility shift assay, and chromatin immunoprecipitation assay.

On the other hand, the 3′UTR refers to a non-coding RNA sequence located downstream of the mRNA coding region, which is generally composed of several nucleotides to hundreds of nucleotides (nt) and is an important binding site sequence for post-transcriptional regulation [66]. MicroRNAs (miRNAs) are a class of small non-coding RNA molecules composed of 21–22 nt, which can complement and pair with the 3′UTR sequence sites on mRNA, thereby inhibiting mRNA translation and regulating gene expression [67,68]. At present, there is insufficient research on how mutations in the 3′UTR of the sheep *FABP4* gene affect the composition of FAs and chemical composition in sheep. However, as has been found in other aspects, such as in tumor research, the rs1054135G>A mutation in the 3′UTR of the *FABP4* gene in triple-negative breast cancer may lead to the enhanced binding of miR-3685 complementary to rs1054135, thereby inhibiting *FABP4* gene expression and reducing fat synthesis [69,70]. In a study on the mutations of g.49400A>G and g.49587A>T in the 3′UTR of the *FGFR1* gene in Tibetan sheep, it was found that the weight of the AA genotype of g.49587A>T was significantly higher than that of the TT genotype. It was directly confirmed that 3′UTR mutations could affect the weight of sheep [71]. According to the results of this study, the g.57757988A>G polymorphism in the 3′UTR of the *FABP4* gene is related to the content of C22:6n3, the live weight, and the carcass weight, and the g.57758026G>A polymorphism is related to the content of Ala; it can therefore be speculated that the g.57757988A>G and g.57758026G>A SNPs in the 3′UTR of the *FABP4* gene can affect the meat quality and carcass traits of Sonid lambs through enhancing the binding ability of the 3′UTR to the corresponding miRNA, weakening the expression of the *FABP4* gene and further leading to changes in FA composition, amino acid content, and carcass traits. Of course, verification tests such as the miRNA species prediction test and luciferase reporter gene assay are still needed to verify the correctness of this inference.

In addition, non-genetic factors have important effects on lamb economic traits. There are other factors that contribute to FAs, such as breed, sex, ram [72], slaughter age [73], and especially feed [74]. It is important to note that among the effects of breeds (including genetic factors), grassland types (including environment and climate), and grazing habits (including local latitude and culture) on the meat FAs of local sheep breeds in a natural grazing system, such as the Mongolian Plateau, the genetic effects, including major genes, are more efficient for improving the meat quality in local sheep breeds. This will make it more practical to meet the demands of consumers who enjoy grazed sheep meat now and in the future. Furthermore, grazing Sonid sheep had higher levels of n-3 in meat than those fed in barns [5], and the grazing Ujimqin sheep (a typical homologous breed of Mongolia sheep) had higher levels of n-3 in serum than those fed in barns [75]. The Sonid lambs in this paper were naturally grazed on the same Sonid grassland after weaning and before slaughter, so there was little difference in feed conditions and living environment, and genetic factors were the most important factors affecting their traits. Our results from the association study may be useful for selecting rams in genetic breeding utilizing markers in the *FABP4* gene.

## 5. Conclusions

In conclusion, nine polymorphisms of *FABP4* were identified in the Sonid sheep breed, including seven mutations in the promoter region and two mutations in the 3′UTR. Based on the results of the association study, these polymorphisms are significantly associated with FA content, chemical composition, and carcass traits. Therefore, the results of these associations provide the possibility for breeders to select sheep with high meat quality and beneficial carcass traits by using the *FABP4* gene as an important molecular marker.

## Figures and Tables

**Table 1:** The primers of *GAPDH* and *FABP4* for real-time quantitative PCR.

Gene	Primer Sequence (5′-3′)	Product Size (bp)	Annealing Temperature (°C)
*GAPDH*	F: TTCCACGGCACAGTCAAGG	114	60.5
	R: CTCAGCACCAGCATCACCC		
*FABP4*	F: AATACTGAGATGTCCTTC	140	53.8
	R: TTTATGGTGGTTGATTTC		

Note: F—forward primer; R—reverse primer.

**Table 2:** Associations of *FABP4* variants with fatty acid composition in *longissimus thoracis* muscles in Sonid sheep.

Fatty Acid Composition (mg/100 g)	g.57765038C>T		g.57765008A>G-LD1		g.57764667T>C		g.57764436T>G		g.57764242G>A			g.57757988A>G	
	Genotype		Genotype		Genotype		Genotype		Genotype			Genotype	
	CT (51) ^1^	TT (216)	AG (49)	GG (218)	TT (245)	TC (25)	TT (253)	TG (18)	GG (89)	GA (133)	AA (49)	AA (247)	AG (23)
C16:1	0.20 ± 0.02 ^a^	0.25 ± 0.01 ^b^	0.20 ± 0.02 ^a^	0.25 ± 0.01 ^b^	0.24 ± 0.01	0.25 ± 0.04	0.24 ± 0.01	0.30 ± 0.04	0.25 ± 0.02	0.24 ± 0.01	0.22 ± 0.02	0.24 ± 0.01	0.25 ± 0.04
C18:1n9t	1.02 ± 0.29	1.04 ± 0.15	1.20 ± 0.30	1.43 ± 0.15	1.37 ± 0.14	1.61 ± 0.45	1.46 ± 0.14 ^a^	0.32 ± 0.04 ^b^	1.18 ± 0.21	1.48 ± 0.20	1.48 ± 0.34	1.38 ± 0.14	1.48 ± 0.45
C18:1n9c	0.56 ± 0.21 ^a^	1.04 ± 0.18 ^b^	0.58 ± 0.22 ^a^	1.40 ± 0.18 ^b^	1.20 ± 0.16	1.65 ± 0.64	1.20 ± 0.16	1.71 ± 0.61	1.71 ± 0.32 ^a^	0.90 ± 0.18 ^b^	1.30 ± 0.37 ^ab^	1.19 ± 0.16	1.78 ± 0.69
C18:2n6c	0.76 ± 0.03	0.76 ± 0.02	0.75 ± 0.03	0.76 ± 0.02	0.75 ± 0.01	0.83 ± 0.06	0.75 ± 0.01 ^a^	0.90 ± 0.07 ^b^	0.80 ± 0.03 ^a^	0.73 ± 0.02 ^b^	0.76 ± 0.03 ^ab^	0.75 ± 0.01	0.84 ± 0.06
C18:3n3	0.21 ± 0.01	0.22 ± 0.00	0.21 ± 0.01	0.22 ± 0.00	0.21 ± 0.00	0.24 ± 0.02	0.21 ± 0.00	0.23 ± 0.01	0.23 ± 0.01 ^a^	0.21 ± 0.01 ^b^	0.21 ± 0.01 ^ab^	0.21 ± 0.00	0.24 ± 0.02
C20:3n3	0.33 ± 0.03	0.28 ± 0.01	0.33 ± 0.03	0.28 ± 0.01	0.28 ± 0.01	0.37 ± 0.04	0.31 ± 0.01 ^a^	0.07 ± 0.04 ^b^	0.28 ± 0.02	0.29 ± 0.02	0.30 ± 0.03	0.28 ± 0.01	0.37 ± 0.04
C22:6n3	0.01 ± 0.00	0.02 ± 0.00	0.01 ± 0.00	0.02 ± 0.00	0.02 ± 0.00 ^a^	0.03 ± 0.00 ^b^	0.02 ± 0.00	0.02 ± 0.00	0.02 ± 0.00	0.02 ± 0.00	0.02 ± 0.00	0.02 ± 0.00 ^a^	0.03 ± 0.00 ^b^

^a,b^ Means that the difference between different superscript values within the same line is statistically significant (*p* < 0.05). ^1^ Represents the mean ± standard error.

**Table 3:** Association of *FABP4* variants with chemical composition of *longissimus thoracis* muscle in Sonid sheep.

Chemical Composition		g.57764436T>G		g.57764242G>A			g.57758026G>A	
		Genotype		Genotype			Genotype	
		TT (92) ^1^	TG (19)	GG (18)	GA (55)	AA (38)	GG (96)	GA (15)
Crude protein (%)		21.16 ± 0.13	20.66 ± 0.33	20.88 ± 0.22 ^a^	21.13 ± 0.17 ^ab^	21.6 ± 0.27 ^b^	21.15 ± 0.12	20.27 ± 0.57
Crude fat (g/100 g)		6.96 ± 0.12	6.94 ± 0.31	7.43 ± 0.21 ^a^	6.75 ± 0.15 ^b^	6.59 ± 0.27 ^b^	6.93 ± 0.12	7.75 ± 0.28
Free amino acid (ng/0.1 g)	Asp	49.66 ± 2.67 ^a^	95.74 ± 58.72 ^b^	83.34 ± 34.68 ^a^	47.08 ± 3.71 ^b^	50.90 ± 4.27 ^ab^	54.33 ± 5.98	42.39 ± 15.99
	Ala	2042.17 ± 35.20	2204.71 ± 279.04	2118.71 ± 162.87	2027.49 ± 48.53	2073.87 ± 61.66	2075.41 ± 40.78 ^a^	1696.45 ± 131.76 ^b^
	Lys	1245.22 ± 28.23 ^a^	1464.63 ± 128.07 ^b^	1348.55 ± 85.35	1235.97 ± 45.24	1268.45 ± 30.58	1255.31 ± 28.99	1404.62 ± 129.27

^a,b^ Means that the difference between different superscript values within the same line is statistically significant (*p* < 0.05). ^1^ Represents the mean ± standard error.

**Table 4:** Association of *FABP4* variants with carcass traits in Sonid sheep.

Carcass Trait ^1^	g.57764667T>C		g.57764242G>A			g.57757988A>G	
	Genotype		Genotype			Genotype	
	TT (95) ^2^	TC (16)	GG (18)	GA (55)	AA (38)	AA (95)	AG (16)
L* (lightness)	23.01 ± 0.31	24.28 ± 0.73	23.22 ± 0.49	23.12 ± 0.39	23.13 ± 0.85	23.01 ± 0.31	24.28 ± 0.73
a* (redness)	13.98 ± 0.27	13.80 ± 0.67	13.75 ± 0.63	14.03 ± 0.27	14.13 ± 0.48	13.98 ± 0.27	13.80 ± 0.67
b* (yellowness)	5.07 ± 0.14	4.93 ± 0.35	5.20 ± 0.22	5.10 ± 0.19	4.69 ± 0.24	5.07 ± 0.14	4.93 ± 0.35
pH_1_ (at 45 min)	6.56 ± 0.03	6.56 ± 0.09	6.55 ± 0.05	6.53 ± 0.03	6.66 ± 0.06	6.56 ± 0.03	6.56 ± 0.09
pH_2_ (at 24 h)	5.41 ± 0.01	5.46 ± 0.05	5.41 ± 0.01	5.43 ± 0.02	5.39 ± 0.02	5.41 ± 0.01	5.46 ± 0.05
Carcass weight (kg)	11.10 ± 0.24 ^a^	9.33 ± 0.80 ^b^	11.84 ± 0.58 ^a^	10.87 ± 0.31 ^ab^	9.89 ± 0.41 ^b^	11.10 ± 0.24 ^a^	9.33 ± 0.80 ^b^
Live weight (kg)	25.67 ± 0.45 ^A^	21.14 ± 1.50 ^B^	26.53 ± 0.93 ^a^	25.15 ± 0.61 ^ab^	23.56 ± 0.97 ^b^	25.67 ± 0.45 ^A^	21.14 ± 1.50 ^B^
Dressing percentage	0.43 ± 0.01	0.44 ± 0.03	0.45 ± 0.02	0.43 ± 0.01	0.42 ± 0.01	0.43 ± 0.01	0.44 ± 0.03

^a,b^ Means that the difference between different superscript values within the same line is statistically significant (*p* < 0.05). ^A,B^ Means that the difference between different superscript values within the same line is statistically significant (*p* < 0.01). ^1^ The L*, a*, b*, pH_1_, and pH_2_ were determined in the *longissimus thoracis* muscle of Sonid sheep. ^2^ Represents the mean ± standard error.

## Data Availability

The original contributions presented in this study are included in the article/Supplementary Materials. Further inquiries can be directed to the corresponding author.

## Supplementary Materials

The following supporting information can be downloaded at https://www.mdpi.com/article/10.3390/ani15020226/s1, Table S1: PCR primers used for sequencing of *FABP4*; Table S2: MassARRAY primers used for the sequencing of the *FABP4* gene; Table S3: Descriptive statistics for fatty acid in *longissimus thoracis* of Sonid sheep; Table S4: Correlation analyses between any two traits of fatty acid compositions and classes; Table S5: Genotypic frequencies, allelic frequencies, and diversity parameters of seven polymorphisms in the Sonid population; Table S6: Linkage disequilibrium as measured by D’ and *r*^2^ among eight polymorphisms in *FABP4*; Table S7: Associations of *FABP4* polymorphisms with carcass traits in Sonid sheep (and continued); Table S8: Associations of *FABP4* polymorphisms with fatty acid composition in *longissimus thoracis* muscle in Sonid sheep (continued); Table S9: Association of *FABP4* polymorphisms with chemical composition in *longissimus thoracis* muscle in Sonid sheep (continued).
