# Supplementary material for: Effect of FABP4 Gene Polymorphisms on Fatty Acid Composition, Chemical Composition, and Carcass Traits in Sonid Sheep
Source: Animals (Basel). 2025 Jan 15;15(2):226. doi: 10.3390/ani15020226 (PMC11758647; doi:10.3390/ani15020226)
Supplement: Supplementary file 1 [file animals-15-00226-s001.zip › Table S1.pdf]

Table S1. PCR primers used for sequencing of *FABP4*.

Name	Target region	Primer sequence (5'-3')	Annealing temperature (°C)	Product size (bp)
Promoter-1	Promoter	F: ACATTACTAAGTGAAAGAAGCC<br>R: TGATCAAGTTTCATACAGGGA	58	762 (762 bp promoter)
Promoter-2	Promoter	F: GCCACAGAGTACATGAGAA<br>R: CAGATCTAAATGATGTTGCTT	56	718 (718 bp promoter)
Promoter-3	Promoter	F: GCCCATGTGACTGTCTTCC<br>R: ACAGCTTAAAATTCAGTGCAT	60	700 (700 bp promoter)
FABP4-1	Exon 1	F: TCCATGACCATTTGCCAAG<br>R: CAGAAAGCCAAGTAAACCA	56	620 (244 bp promoter + 73 bp exon 1 + 303 bp intron 1)
FABP4-2	Exon 2	F: GAGAAATTTAAAGAACAGAC<br>R: ATTTTTAAAGTAATAAGGACA	56	629 (251 bp intron 1 + 173 bp exon 2 + 205 bp intron 2)
FABP4-3	Exon 3	F: TATGTATATTCTTTCACGGTA<br>R: AAAACATAATTAGAAATGCAC	56	559 (218 bp intron 2 + 102 bp exon 3 + 239 bp intron 3)
FABP4-4	Exons 4	F: CTCTGTCTATGTATATTGTGC<br>R: TACGACTCATATTACACTGG	58	642 (313 bp intron 3 + 51 bp exon 4 + 278 bp 3'UTR)
FABP4-3'UTR	3'UTR	F: AATATTTTGCCTTTGTGCCTA<br>R: AGCCTGTCATGTCTTTTCTG	60	731 (22 bp intron 3 + 51 bp exon 4 + 500 bp 3'UTR + 158 bp)

PCR reaction assays were set in 50 μL total volume containing 1 μM of each primer, 0.5 μL of TaKaRa LA Taq® (Takara, Dalian, China), 5 μL of 10 x LA Taq Buffer, 8 μL of dNTP mix, 32.5 μL of ddH_2O, and 2 μL of DNA template.
