# Supplementary material for: Effect of FABP4 Gene Polymorphisms on Fatty Acid Composition, Chemical Composition, and Carcass Traits in Sonid Sheep
Source: Animals (Basel). 2025 Jan 15;15(2):226. doi: 10.3390/ani15020226 (PMC11758647; doi:10.3390/ani15020226)
Supplement: Supplementary file 1 [file animals-15-00226-s001.zip › Table S2.pdf]

Table S2. MassARRAY primers were used for sequencing of the *FABP4* gene.

Variant	Primer (5′-3′) [1]	Tm (°C)
g.57765038C>T	F: ACGTTGGATGATGAATAGGCAAAGCCAGAG<br>R: ACGTTGGATGGGTGGTTTGCCATTCTTCC<br>E: AAAGCCAGAGGGTTTT	57.6
g.57765008A>G	F: ACGTTGGATGGGTGGTTTGCCATTCTTCC<br>R: ACGTTGGATGATGAATAGGCAAAGCCAGAG<br>E: TCATGACATATATTCATCCT	51.2
g.57764906T>C	F: ACGTTGGATGATGAATAGGCAAAGCCAGAG<br>R: ACGTTGGATGGGTGGTTTGCCATTCTTCC<br>E: CTGGGTCAGGAAGAT	55.4
g.57764667T>C	F: ACGTTGGATGTCTCATGTACTCTGTGGCTG<br>R: ACGTTGGATGCCCTAAGGTAATATATGGAC<br>E: AAGAATGGTAATTTTTACT	50.1
g.57764632A>G	F: ACGTTGGATGCCCTAAGGTAATATATGGAC<br>R: ACGTTGGATGTCTCATGTACTCTGTGGCTG<br>E: GTCCCATTCTGGTGAATGATATTGAT	61.2
g.57764436T>G	F: ACGTTGGATGCAAACTAAAAACAAAAGCC<br>R: ACGTTGGATGTGACGTACCATGCAGGGTAG<br>E: AAATTGATCATTTTTATTTTCTTGT	55.2
g.57764242G>A	F: ACGTTGGATGGCTTCCCAGAAATGGTTGAG<br>R: ACGTTGGATGTGTGACACAACATTACCAG<br>E: GAAAGGGTTGAGTAAGAGGATTC	56.2
g.57758026G>A	F: ACGTTGGATGAAGAGAGGTGGAATAGGAAC<br>R: ACGTTGGATGCCAGGAATACCTTGGTTTAC<br>E: GTGGCATAGGAACAAAGAGT	48.3
g.57757988A>G	F: ACGTTGGATGAAGAGAGGTGGAATAGGAAC<br>R: ACGTTGGATGCCAGGAATACCTTGGTTTAC<br>E: TGGTCTATAAATTTTAGCCTGAA	51.9

[1] E: Extended primer sequence.
