# Supplementary material for: Effect of FABP4 Gene Polymorphisms on Fatty Acid Composition, Chemical Composition, and Carcass Traits in Sonid Sheep
Source: Animals (Basel). 2025 Jan 15;15(2):226. doi: 10.3390/ani15020226 (PMC11758647; doi:10.3390/ani15020226)
Supplement: Supplementary file 1 [file animals-15-00226-s001.zip › Table S3.pdf]

Table S3. Descriptive statistics for the FA in *longissimus thoracis* of Sonid sheep.

Fatty acid	Nomenclature	Mean [1]	SD [2]
Butryic acid	C4:0	4.11	0.99
Caprylic acid	C8:0	171.64	23.25
Capric acid	C10:0	22.23	3.00
Lauric acid	C12:0	6.29	1.22
Myristic acid	C14:0	41.62	2.19
Pentadecanoic acid	C15:0	16.80	1.30
Palmitic acid	C16:0	222.29	13.10
Heptadecanoic acid	C17:0	20.42	1.00
Stearic acid	C18:0	251.04	7.54
Arachidic acid	C20:0	2.04	0.99
Heneicosylic acid	C21:0	1.40	0.99
Behenic acid	C22:0	7.06	0.99
Tricosanoic acid	C23:0	43.91	1.58
Tetracosanoic acid	C24:0	9.40	0.97
Saturated fatty acid	SFA	617.06	22.46
Myristoleic acid	C14:1	3.39	1.00
Pentadecenoic acid	C15:1	19.24	5.17
Palmitoleic acid	C16:1	25.45	1.33
Ginkgolic acid	C17:1	23.74	3.11
Elaidic acid	C18:1n9t	138.97	13.27
Oleic acid	C18:1n9c	123.87	15.30
cis-11-Eicosenopc	C20:1	2.64	1.17
Nervonic acid	C24:1	1.82	0.98
Monounsaturated fatty acid	MUFA	325.49	21.63
Linolelaidic acid	C18:2n6t	3.65	1.03
Linoleic acid	C18:2n6c	77.76	1.50
Gamma-linolenic acid	C18:3n6	1.32	0.99
α-Linolenic acid	C18:3n3	22.48	0.99
Conjugated linoleic acid	C18:2c9t11	18.69	1.16
11C,14C-Eicosadienoic acid	C20:2n6	9.19	0.98
dihomo-γ-linolenic acid	C20:3n6	3.43	0.98
Cis-11,14,17-Eicosatrienoic acid	C20:3n3	43.10	0.95
Arachidonic acid	C20:4n6	2.38	0.99
13C,16C-Docosadienoic acid	C22:2	3.73	0.97
Cis-5,8,11,14,17-Eicosapentaenoic acid	C20:5n3	10.18	0.78
Docosahexaaonic acid	C22:6n3	2.72	1.00
Polyunsaturated fatty acid	PUFA	156.28	2.28
Unsaturated fatty acid	UFA	481.77	22.34
MUFA/SFA	MUFA/SFA	0.70	0.07
PUFA/SFA	PUFA/SFA	0.35	0.01
UFA/SFA	UFA/SFA	1.05	0.08
Short chain fatty acid	SCFA	3.13	0.10
Medium chain fatty acid	MCFA	45.62	4.88
Long chain fatty acid	LCFA	1050.08	34.51
Omega 6 Polyunsaturated fatty acids	n-6	102.36	1.95
Omega 3 Polyunsaturated fatty acids	n-3	52.95	1.19
n-6/n-3	n-6/n-3	3.09	0.72
Essential fatty acid	EFA	155.31	2.27

[1] Mean is the mean value. [2] SD is the standard deviation.
