# Supplementary material for: Effect of FABP4 Gene Polymorphisms on Fatty Acid Composition, Chemical Composition, and Carcass Traits in Sonid Sheep
Source: Animals (Basel). 2025 Jan 15;15(2):226. doi: 10.3390/ani15020226 (PMC11758647; doi:10.3390/ani15020226)
Supplement: Supplementary file 1 [file animals-15-00226-s001.zip › Table S4.pdf]

Table S4. Correlation analyses between any two traits of FA compositions and classes.

**Fatty acid**	**C4:0**	**C8:0**	**C10:0**	**C12:0**	**C14:0**	**C15:0**	**C16:0**	**C17:0**	**C18:0**	**C20:0**
C4:0	1.000	-0.586**	-0.215**	-0.027	-0.035	-0.196**	0.120*	0.024	-0.006	0.047
C8:0	-0.586** [1]	1.000	-0.341	-0.196	0.162	-0.346	0.672**	0.022	0.059	0.400*
C10:0	-0.215**	-0.341	1.000	-0.071	-0.039	0.489**	-0.376**	0.196**	0.042	-0.156*
C12:0	-0.027	-0.196	-0.071	1.000	0.089	0.030	0.001	-0.061	0.025	0.039
C14:0	-0.035	0.162	-0.039	0.089	1.000	-0.040	0.670**	0.500**	0.930**	0.543**
C15:0	-0.196**	-0.346	0.489**	0.030	-0.040	1.000	-0.557**	0.447**	0.026	-0.150*
C16:0	0.120* [2]	0.672**	-0.376**	0.001	0.670**	-0.557**	1.000	0.225**	0.628**	0.585**
C17:0	0.024	0.022	0.196**	-0.061	0.500**	0.447**	0.225**	1.000	0.552**	0.353**
C18:0	-0.006	0.059	0.042	0.025	0.930**	0.026	0.628**	0.552**	1.000	0.579**
C20:0	0.047	0.400*	-0.156*	0.039	0.543**	-0.150*	0.585**	0.353**	0.579**	1.000
C21:0	0.032	-0.544	-0.111	0.523**	0.559**	0.126	0.248*	0.348**	0.599**	-0.045
C22:0	0.276**	0.678**	-0.457**	-0.062	0.410**	-0.714**	0.798**	-0.044	0.366**	0.466**
C23:0	0.172	0.426*	-0.305**	0.234*	0.270*	-0.038	0.325**	0.295**	0.302**	0.451**
C24:0	0.063	-0.171	0.116	-0.294**	0.145*	0.116	0.070	0.380**	0.191**	0.073
C14:1	-0.173**	-0.275	0.361**	0.040	0.538**	0.529**	-0.204**	0.392**	0.574**	0.133
C15:1	-0.155*	-0.243	0.298**	-0.020	0.059	0.213**	-0.194**	0.079	0.056	0.010
C16:1	-0.087	0.545**	0.035	-0.018	0.510**	-0.020	0.443**	0.331**	0.469**	0.448**
C17:1	-0.154*	-0.236	0.671**	-0.049	0.001	0.240**	-0.156*	0.122	0.042	0.053
C18:1n9t	-0.237**	-0.356*	0.732**	-0.012	0.053	0.626**	-0.461**	0.275**	0.108	-0.123
C18:1n9c	0.014	0.384*	-0.155*	0.011	0.397**	-0.215**	0.522**	0.169**	0.401**	0.484**
C20:1	-0.115	-0.376	-0.250	-0.126	-0.193	-0.224	-0.223	-0.262	-0.268	-0.150
C24:1	0.103	-0.534	-0.106	-0.145	-0.155	0.377**	-0.253*	0.329**	-0.094	-0.143
C18:2n6t	-0.029	0.356*	-0.080	-0.007	0.322**	-0.118	0.206**	0.107	0.194**	0.182**
C18:2n6c	0.024	0.221	-0.050	-0.101	0.630**	0.071	0.445**	0.464**	0.664**	0.533**
C18:3n6	-0.148	-0.299	0.317**	0.064	0.162	0.142	-0.076	0.073	0.185*	0.107
C18:3n3	0.025	0.183	0.012	-0.118	0.790**	0.012	0.562**	0.536**	0.823**	0.496**

C18:2c9t11	-0.054	0.242	-0.020	0.040	0.917**	-0.085	0.695**	0.467**	0.890**	0.574**
C20:2n6	-0.109	-0.273	0.206*	-0.170	-0.329**	0.425**	-0.732**	-0.199*	-0.253**	-0.385**
C20:3n6	0.052	-0.041	0.037	-0.094	0.320**	0.066	0.318**	0.320**	0.343**	0.347**
C20:3n3	-0.041	-0.212	0.056	-0.327**	0.069	0.476**	-0.293**	0.530**	0.127	0.035
C20:4n6	0.348*	0.000	-0.211	-0.114	0.190	-0.408**	0.318*	-0.160	0.180	0.226
C22:2	0.198	0.350	0.170	-0.023	0.186	0.200	0.226*	0.204*	0.215*	0.095
C20:5n3	0.429*	0.000	-0.756**	0.173	0.383	-0.545**	0.479*	0.120	0.197	0.435*
C22:6n3	-0.019	-0.149	0.021	-0.005	0.134*	0.048	0.042	0.232**	0.107	0.052
SFA	0.011	0.675**	-0.133*	0.041	0.868**	-0.294**	0.896**	0.409**	0.855**	0.620**
MUFA	-0.189**	0.166	0.416**	-0.008	0.355**	0.291**	0.055	0.336**	0.413**	0.351**
PUFA	0.023	-0.088	0.035	-0.107	0.633**	0.259**	0.261**	0.605**	0.666**	0.394**
UFA	-0.181**	0.147	0.406**	-0.018	0.409**	0.309**	0.080	0.387**	0.472**	0.386**
MUFA/SFA	-0.161**	-0.237	0.366**	-0.036	-0.081	0.406**	-0.324**	0.155*	-0.043	-0.046
PUFA/SFA	0.020	-0.703**	0.061	-0.079	-0.500**	0.458**	-0.663**	0.101	-0.544**	-0.428**
UFA/SFA	-0.139*	-0.309	0.336**	-0.047	-0.165**	0.447**	-0.411**	0.157**	-0.182**	-0.139*
SCFA	1.000**	-0.586**	-0.215**	-0.027	-0.035	-0.196**	0.120*	0.024	-0.006	0.047
MCFA	-0.257**	0.979**	0.481**	0.101	0.160**	0.144*	0.003	0.096	0.124*	-0.016
LCFA	-0.076	0.376*	0.102	0.000	0.807**	-0.012	0.634**	0.503**	0.820**	0.643**
n-6	0.021	0.225	-0.107	-0.057	0.783**	0.007	0.548**	0.515**	0.779**	0.603**
n-3	0.009	-0.720**	0.263**	-0.112	-0.088	0.518**	-0.437**	0.327**	-0.017	-0.258**
n-6/n-3	-0.061	0.080	-0.055	-0.020	0.028	-0.096	0.065	-0.095	-0.005	0.039
EFA	0.022	-0.100	0.045	-0.106	0.625**	0.276**	0.242**	0.612**	0.661**	0.391**

[1] ***p* < 0.01. [2] **p* < 0.05.

Table S4. (Continue)

Fatty acid	C21:0	C22:0	C23:0	C24:0	C14:1	C15:1	C16:1	C17:1	C18:1n9t	C18:1n9c
C4:0	0.032	0.276**	0.172	0.063	-0.173**	-0.155*	-0.087	-0.154*	-0.237**	0.014
C8:0	-0.544	0.678**	0.426*	-0.171	-0.275	-0.243	0.545**	-0.236	-0.356*	0.384*
C10:0	-0.111	-0.457**	-0.305**	0.116	0.361**	0.298**	0.035	0.671**	0.732**	-0.155*
C12:0	0.523** [1]	-0.062	0.234*	-0.294**	0.040	-0.020	-0.018	-0.049	-0.012	0.011
C14:0	0.559**	0.410**	0.270*	0.145*	0.538**	0.059	0.510**	0.001	0.053	0.397**
C15:0	0.126	-0.714**	-0.038	0.116	0.529**	0.213**	-0.020	0.240**	0.626**	-0.215**
C16:0	0.248* [2]	0.798**	0.325**	0.070	-0.204**	-0.194**	0.443**	-0.156*	-0.461**	0.522**
C17:0	0.348**	-0.044	0.295**	0.380**	0.392**	0.079	0.331**	0.122	0.275**	0.169**
C18:0	0.599**	0.366**	0.302**	0.191**	0.574**	0.056	0.469**	0.042	0.108	0.401**
C20:0	-0.045	0.466**	0.451**	0.073	0.133	0.010	0.448**	0.053	-0.123	0.484**
C21:0	1.000	0.042	-0.091	0.153	0.416**	0.160	-0.073	0.118	0.024	-0.052
C22:0	0.042	1.000	0.359**	0.157*	-0.283**	-0.183**	0.288**	-0.175*	-0.568**	0.422**
C23:0	-0.091	0.359**	1.000	0.222	0.132	-0.027	0.346**	0.019	-0.142	-0.080
C24:0	0.153	0.157*	0.222	1.000	0.110	0.109	0.150*	0.059	0.141*	-0.160*
C14:1	0.416**	-0.283**	0.132	0.110	1.000	0.317**	0.187**	0.446**	0.577**	-0.006
C15:1	0.160	-0.183**	-0.027	0.109	0.317**	1.000	0.157*	0.976**	0.478**	-0.085
C16:1	-0.073	0.288**	0.346**	0.150*	0.187**	0.157*	1.000	0.091	0.104	0.507**
C17:1	0.118	-0.175*	0.019	0.059	0.446**	0.976**	0.091	1.000	0.768**	-0.053
C18:1n9t	0.024	-0.568**	-0.142	0.141*	0.577**	0.478**	0.104	0.768**	1.000	-0.200**
C18:1n9c	-0.052	0.422**	-0.080	-0.160*	-0.006	-0.085	0.507**	-0.053	-0.200**	1.000
C20:1	0.278	-0.292	-0.183	-0.163	-0.185	-0.284	-0.191	-0.256	0.002	-0.441*
C24:1	-0.139	-0.236*	-0.967*	0.086	-0.045	-0.108	-0.101	-0.081	0.029	-0.091
C18:2n6t	0.513**	0.224**	0.011	0.018	0.200**	0.294**	0.147*	0.443**	0.120*	0.120*
C18:2n6c	0.243*	0.291**	0.759**	0.212**	0.349**	0.008	0.446**	-0.043	-0.021	0.324**
C18:3n6	-0.174	-0.152	0.224	-0.085	0.222*	-0.019	0.084	0.173	0.398**	-0.114
C18:3n3	0.463**	0.433**	0.387**	0.476**	0.434**	0.133*	0.495**	0.096	0.119*	0.339**
C18:2c9t11	0.601**	0.475**	0.165	0.211**	0.457**	0.155*	0.540**	0.124	0.106	0.411**

C20:2n6	-0.201	-0.762**	-0.715**	-0.065	0.288**	0.077	-0.355**	0.053	0.302**	-0.507**
C20:3n6	0.058	0.469**	0.580**	0.294**	0.156	0.005	0.300**	0.074	0.072	0.255**
C20:3n3	0.008	-0.356**	-1.000**	0.257**	0.303**	0.046	0.000	0.053	0.182*	-0.012
C20:4n6	-0.343	0.351*	-0.292	-0.323	-0.263	-0.169	0.237	-0.218	-0.269	0.482**
C22:2	0.141	0.254*	0.404**	0.335**	0.123	0.337**	0.159	0.173	0.184	-0.199
C20:5n3	-0.649*	0.814**	0.107	-0.988	0.264	0.138	0.432*	-0.678**	-0.733**	0.512*
C22:6n3	0.264**	0.004	0.372**	0.328**	0.140*	0.002	0.083	0.098	0.032	-0.026
SFA	0.369**	0.644**	0.282**	0.106	0.151*	-0.125*	0.530**	-0.140*	-0.190**	0.539**
MUFA	0.032	-0.086	-0.089	0.045	0.464**	0.553**	0.516**	0.549**	0.621**	0.588**
PUFA	0.495**	0.070	0.589**	0.334**	0.561**	0.178**	0.343**	0.144*	0.197**	0.099
UFA	0.074	-0.076	-0.025	0.080	0.506**	0.553**	0.535**	0.549**	0.621**	0.579**
MUFA/SFA	0.087	-0.356**	-0.133	0.042	0.299**	0.693**	0.103	0.895**	0.627**	0.021
PUFA/SFA	-0.040	-0.583**	0.042	0.073	0.078	0.268**	-0.294**	0.383**	0.243**	-0.359**
UFA/SFA	0.072	-0.410**	-0.122	0.050	0.279**	0.666**	0.037	0.864**	0.603**	-0.048
SCFA	0.032	0.276**	0.172	0.063	-0.173**	-0.155*	-0.087	-0.154*	-0.237**	0.014
MCFA	-0.151	-0.144*	-0.174	-0.095	0.146*	0.100	0.185**	0.128	0.288**	0.132*
LCFA	0.327**	0.394**	0.179	0.146*	0.412**	0.264**	0.665**	0.214**	0.239**	0.707**
n-6	0.429**	0.375**	0.658**	0.207**	0.428**	0.132*	0.486**	0.111	0.020	0.378**
n-3	0.224*	-0.518**	0.151	0.298**	0.391**	0.137*	-0.150*	0.097	0.369**	-0.417**
n-6/n-3	0.013	0.044	-0.138	-0.119	-0.059	-0.017	0.016	-0.020	-0.063	0.101
EFA	0.489**	0.050	0.577**	0.329**	0.572**	0.184**	0.339**	0.150*	0.210**	0.107

[1] ** $p < 0.01$. [2] * $p < 0.05$.

Table S4. (Continue)

Fatty acid	C20:1	C24:1	C18:2n6t	C18:2n6c	C18:3n6	C18:3n3	C18:2c9t11	C20:2n6	C20:3n6	C20:3n3
C4:0	-0.115	0.103	-0.029	0.024	-0.148	0.025	-0.054	-0.109	0.052	-0.041
C8:0	-0.376	-0.534	0.356*	0.221	-0.299	0.183	0.242	-0.273	-0.041	-0.212
C10:0	-0.250	-0.106	-0.080	-0.050	0.317**	0.012	-0.020	0.206*	0.037	0.056
C12:0	-0.126	-0.145	-0.007	-0.101	0.064	-0.118	0.040	-0.170	-0.094	-0.327**
C14:0	-0.193	-0.155	0.322**	0.630**	0.162	0.790**	0.917**	-0.329**	0.320**	0.069
C15:0	-0.224	0.377** [2]	-0.118	0.071	0.142	0.012	-0.085	0.425**	0.066	0.476**
C16:0	-0.223	-0.253*	0.206**	0.445**	-0.076	0.562**	0.695**	-0.732**	0.318**	-0.293**
C17:0	-0.262	0.329**	0.107	0.464**	0.073	0.536**	0.467**	-0.199*	0.320**	0.530**
C18:0	-0.268	-0.094	0.194**	0.664**	0.185*	0.823**	0.890**	-0.253**	0.343**	0.127
C20:0	-0.150	-0.143	0.182**	0.533**	0.107	0.496**	0.574**	-0.385**	0.347**	0.035
C21:0	0.278	-0.139	0.513**	0.243*	-0.174	0.463**	0.601**	-0.201	0.058	0.008
C22:0	-0.292	-0.236*	0.224**	0.291**	-0.152	0.433**	0.475**	-0.762**	0.469**	-0.356**
C23:0	-0.183	-0.967*	0.011	0.759**	0.224	0.387**	0.165	-0.715**	0.580**	-1.000**
C24:0	-0.163	0.086	0.018	0.212**	-0.085	0.476**	0.211**	-0.065	0.294**	0.257**
C14:1	-0.185	-0.045	0.200**	0.349**	0.222*	0.434**	0.457**	0.288**	0.156	0.303**
C15:1	-0.284	-0.108	0.294**	0.008	-0.019	0.133*	0.155*	0.077	0.005	0.046
C16:1	-0.191	-0.101	0.147*	0.446**	0.084	0.495**	0.540**	-0.355**	0.300**	0.000
C17:1	-0.256	-0.081	0.443**	-0.043	0.173	0.096	0.124	0.053	0.074	0.053
C18:1n9t	0.002	0.029	0.120*	-0.021	0.398**	0.119*	0.106	0.302**	0.072	0.182*
C18:1n9c	-0.441* [1]	-0.091	0.120*	0.324**	-0.114	0.339**	0.411**	-0.507**	0.255**	-0.012
C20:1	1.000	0.000	-0.106	-0.275	0.631*	-0.322	-0.400*	0.434	-0.395*	0.000
C24:1	0.000	1.000	-0.169	-0.039	-0.047	-0.064	-0.161	0.016	-0.272	0.430**
C18:2n6t	-0.106	-0.169	1.000	0.009	0.030	0.263**	0.335**	-0.367**	0.102	0.045
C18:2n6c	-0.275	-0.039	0.009	1.000	0.027	0.733**	0.550**	-0.094	0.525**	0.666**
C18:3n6	0.631*	-0.047	0.030	0.027	1.000	0.042	0.125	0.261*	0.008	0.034
C18:3n3	-0.322	-0.064	0.263**	0.733**	0.042	1.000	0.808**	-0.240**	0.501**	0.293**
C18:2c9t11	-0.400*	-0.161	0.335**	0.550**	0.125	0.808**	1.000	-0.364**	0.336**	-0.025

C20:2n6	0.434	0.016	-0.367**	-0.094	0.261*	-0.240**	-0.364**	1.000	-0.262*	0.489**
C20:3n6	-0.395*	-0.272	0.102	0.525**	0.008	0.501**	0.336**	-0.262*	1.000	0.332**
C20:3n3	0.000	0.430**	0.045	0.666**	0.034	0.293**	-0.025	0.489**	0.332**	1.000
C20:4n6	0.021	0.398	0.262	0.363**	-0.227	0.251	0.198	-0.654**	0.272	-0.101
C22:2	-0.367	-0.701	0.195	0.226*	0.091	0.299**	0.201*	-0.664*	0.342**	-0.055
C20:5n3	-0.006	0.000	0.300	0.415*	-0.377	0.516*	0.386	-0.911**	0.136	0.000
C22:6n3	-0.102	-0.031	0.008	0.219**	-0.155	0.265**	0.129*	0.135	0.302**	0.191**
SFA	-0.217	-0.195	0.234**	0.596**	0.043	0.724**	0.845**	-0.559**	0.309**	-0.101
MUFA	-0.412*	-0.036	0.287**	0.239**	0.200*	0.379**	0.433**	-0.053	0.267**	0.153*
PUFA	-0.396*	-0.032	0.160**	0.787**	0.169	0.754**	0.607**	0.070	0.500**	0.568**
UFA	-0.422*	-0.038	0.294**	0.310**	0.210*	0.443**	0.482**	-0.044	0.315**	0.207**
MUFA/SFA	-0.571**	-0.019	0.289**	-0.064	0.115	0.032	0.044	0.140	0.098	0.130
PUFA/SFA	-0.325	0.139	-0.113	-0.200**	-0.069	-0.321**	-0.471**	0.383**	-0.054	0.410**
UFA/SFA	-0.595**	0.003	0.237**	-0.094	0.090	-0.031	-0.048	0.193*	0.076	0.187*
SCFA	-0.115	0.103	-0.029	0.024	-0.148	0.025	-0.054	-0.109	0.052	-0.041
MCFA	0.157	-0.152	0.001	0.072	0.137	0.067	0.143*	0.155	-0.113	-0.020
LCFA	-0.345	-0.109	0.343**	0.580**	0.155	0.748**	0.842**	-0.390**	0.382**	0.109
n-6	-0.350	-0.140	0.273**	0.916**	0.064	0.802**	0.760**	-0.134	0.558**	0.412**
n-3	-0.436*	0.288**	-0.146*	0.033	0.240**	0.101	-0.103	0.612**	0.010	0.858**
n-6/n-3	0.526**	-0.260**	0.025	-0.022	-0.160	0.291**	0.021	-0.558**	-0.037	-0.140
EFA	-0.395*	-0.026	0.158**	0.780**	0.171	0.745**	0.597**	0.085	0.497**	0.579**

[1] ** $p < 0.01$. [2] * $p < 0.05$.

Table S4. (Continue)

Fatty acid	C20:4n6	C22:2	C20:5n3	C22:6n3	SFA	MUFA	PUFA	UFA	MUFA/SFA	PUFA/SFA
C4:0	0.348* [1]	0.198	0.429*	-0.019	0.011	-0.189**	0.023	-0.181**	-0.161**	0.020
C8:0	0.000	0.350	0.000	-0.149	0.675**	0.166	-0.088	0.147	-0.237	0-.703**
C10:0	-0.211	0.170	-0.756**	0.021	-0.133*	0.416**	0.035	0.406**	0.366**	0.061
C12:0	-0.114	-0.023	0.173	-0.005	0.041	-0.008	-0.107	-0.018	-0.036	-0.079
C14:0	0.190	0.186	0.383	0.134*	0.868**	0.355**	0.633**	0.409**	-0.081	-0.500**
C15:0	-0.408** [2]	0.200	-0.545**	0.048	-0.294**	0.291**	0.259**	0.309**	0.406**	0.458**
C16:0	0.318*	0.226*	0.479*	0.042	0.896**	0.055	0.261**	0.080	-0.324**	-0.663**
C17:0	-0.160	0.204*	0.120	0.232**	0.409**	0.336**	0.605**	0.387**	0.155*	0.101
C18:0	0.180	0.215*	0.197	0.107	0.855**	0.413**	0.666**	0.472**	-0.043	-0.544**
C20:0	0.226	0.095	0.435*	0.052	0.620**	0.351**	0.394**	0.386**	-0.046	-0.428**
C21:0	-0.343	0.141	-0.649*	0.264**	0.369**	0.032	0.495**	0.074	0.087	-0.040
C22:0	0.351*	0.254*	0.814**	0.004	0.644**	-0.086	0.070	-0.076	-0.356**	-0.583**
C23:0	-0.292	0.404**	0.107	0.372**	0.282**	-0.089	0.589**	-0.025	-0.133	0.042
C24:0	-0.323	0.335**	-0.988	0.328**	0.106	0.045	0.334**	0.080	0.042	0.073
C14:1	-0.263	0.123	0.264	0.140*	0.151*	0.464**	0.561**	0.506**	0.299**	0.078
C15:1	-0.169	0.337**	0.138	0.002	-0.125*	0.553**	0.178**	0.553**	0.693**	0.268**
C16:1	0.237	0.159	0.432*	0.083	0.530**	0.516**	0.343**	0.535**	0.103	-0.294**
C17:1	-0.218	0.173	-0.678**	0.098	-0.140*	0.549**	0.144*	0.549**	0.895**	0.383**
C18:1n9t	-0.269	0.184	-0.733**	0.032	-0.190**	0.621**	0.197**	0.621**	0.627**	0.243**
C18:1n9c	0.482**	-0.199	0.512*	-0.026	0.539**	0.588**	0.099	0.579**	0.021	-0.359**
C20:1	0.021	-0.367	-0.006	-0.102	-0.217	-0.412*	-0.396*	-0.422*	-0.571**	-0.325
C24:1	0.398	-0.701	0.000	-0.031	-0.195	-0.036	-0.032	-0.038	-0.019	0.139
C18:2n6t	0.262	0.195	0.300	0.008	0.234**	0.287**	0.160**	0.294**	0.289**	-0.113
C18:2n6c	0.363**	0.226*	0.415*	0.219**	0.596**	0.239**	0.787**	0.310**	-0.064	-0.200**
C18:3n6	-0.227	0.091	-0.377	-0.155	0.043	0.200*	0.169	0.210*	0.115	-0.069
C18:3n3	0.251	0.299**	0.516*	0.265**	0.724**	0.379**	0.754**	0.443**	0.032	-0.321**
C18:2c9t11	0.198	0.201*	0.386	0.129*	0.845**	0.433**	0.607**	0.482**	0.044	-0.471**

C20:2n6	-0.654**	-0.664*	-0.911**	0.135	-0.559**	-0.053	0.070	-0.044	0.140	0.383**
C20:3n6	0.272	0.342**	0.136	0.302**	0.309**	0.267**	0.500**	0.315**	0.098	-0.054
C20:3n3	-0.101	-0.055	0.000	0.191**	-0.101	0.153*	0.568**	0.207**	0.130	0.410**
C20:4n6	1.000	-0.442	0.382	-0.092	0.284*	0.344*	0.190	0.344*	-0.068	-0.348*
C22:2	-0.442	1.000	0.000	0.222*	0.194	-0.148	0.279**	-0.091	-0.280**	-0.060
C20:5n3	0.382	0.000	1.000	0.195	0.432*	0.335	0.391	0.345	-0.669**	-0.612**
C22:6n3	-0.092	0.222*	0.195	1.000	0.084	0.017	0.280**	0.044	0.050	0.116
SFA	0.284*	0.194	0.432*	0.084	1.000	0.251**	0.430**	0.286**	-0.253**	-0.700**
MUFA	0.344*	-0.148	0.335	0.017	0.251**	1.000	0.263**	0.995**	0.663**	-0.015
PUFA	0.190	0.279**	0.391	0.280**	0.430**	0.263**	1.000	0.357**	0.099	0.061
UFA	0.344*	-0.091	0.345	0.044	0.286**	0.995**	0.357**	1.000	0.652**	-0.008
MUFA/SFA	-0.068	-0.280**	-0.669**	0.050	-0.253**	0.663**	0.099	0.652**	1.000	0.525**
PUFA/SFA	-0.348*	-0.060	-0.612**	0.116	-0.700**	-0.015	0.061	-0.008	0.525**	1.000
UFA/SFA	-0.128	-0.304**	-0.687**	0.066	-0.355**	0.587**	0.100	0.579**	0.987**	0.653**
SCFA	0.348*	0.198	0.429*	-0.019	0.011	-0.189**	0.023	-0.181**	-0.161**	0.020
MCFA	-0.233	-0.086	-0.745**	0.030	0.276**	0.309**	-0.081	0.291**	0.134*	-0.217**
LCFA	0.351*	0.120	0.430*	0.079	0.797**	0.764**	0.522**	0.793**	0.239**	-0.430**
n-6	0.351*	0.253*	0.422*	0.197**	0.701**	0.348**	0.860**	0.425**	0.037	-0.225**
n-3	-0.338*	0.052	0.071	0.206**	-0.354**	-0.039	0.504**	0.014	0.155*	0.514**
n-6/n-3	0.354*	-0.181	0.253	-0.056	0.077	0.031	-0.143*	0.015	-0.018	-0.125*
EFA	0.201	0.244*	0.391	0.279**	0.416**	0.278**	0.999**	0.371**	0.112	0.075

[1] ** $p < 0.01$. [2] * $p < 0.05$.

Table S4. (Continue)

Fatty acid	UFA/SFA	SCFA	MCFA	LCFA	n-6	n-3	n-6/n-3	EFA
C4:0	-0.139* [1]	1.000**	-0.257**	-0.076	0.021	0.009	-0.061	0.022
C8:0	-0.309	-0.586**	0.979**	0.376*	0.225	-0.720**	0.080	-0.100
C10:0	0.336** [2]	-0.215**	0.481**	0.102	-0.107	0.263**	-0.055	0.045
C12:0	-0.047	-0.027	0.101	0.000	-0.057	-0.112	-0.020	-0.106
C14:0	-0.165**	-0.035	0.160**	0.807**	0.783**	-0.088	0.028	0.625**
C15:0	0.447**	-0.196**	0.144*	-0.012	0.007	0.518**	-0.096	0.276**
C16:0	-0.411**	0.120*	0.003	0.634**	0.548**	-0.437**	0.065	0.242**
C17:0	0.157**	0.024	0.096	0.503**	0.515**	0.327**	-0.095	0.612**
C18:0	-0.182**	-0.006	0.124*	0.820**	0.779**	-0.017	-0.005	0.661**
C20:0	-0.139*	0.047	-0.016	0.643**	0.603**	-0.258**	0.039	0.391**
C21:0	0.072	0.032	-0.151	0.327**	0.429**	0.224*	0.013	0.489**
C22:0	-0.410**	0.276**	-0.144*	0.394**	0.375**	-0.518**	0.044	0.050
C23:0	-0.122	0.172	-0.174	0.179	0.658**	0.151	-0.138	0.577**
C24:0	0.050	0.063	-0.095	0.146*	0.207**	0.298**	-0.119	0.329**
C14:1	0.279**	-0.173**	0.146*	0.412**	0.428**	0.391**	-0.059	0.572**
C15:1	0.666**	-0.155*	0.100	0.264**	0.132*	0.137*	-0.017	0.184**
C16:1	0.037	-0.087	0.185**	0.665**	0.486**	-0.150*	0.016	0.339**
C17:1	0.864**	-0.154*	0.128	0.214**	0.111	0.097	-0.020	0.150*
C18:1n9t	0.603**	-0.237**	0.288**	0.239**	0.020	0.369**	-0.063	0.210**
C18:1n9c	-0.048	0.014	0.132*	0.707**	0.378**	-0.417**	0.101	0.107
C20:1	-0.595**	-0.115	0.157	-0.345	-0.350	-0.436*	0.526**	-0.395*
C24:1	0.003	0.103	-0.152	-0.109	-0.140	0.288**	-0.260**	-0.026
C18:2n6t	0.237**	-0.029	0.001	0.343**	0.273**	-0.146*	0.025	0.158**
C18:2n6c	-0.094	0.024	0.072	0.580**	0.916**	0.033	-0.022	0.780**
C18:3n6	0.090	-0.148	0.137	0.155	0.064	0.240**	-0.160	0.171
C18:3n3	-0.031	0.025	0.067	0.748**	0.802**	0.101	0.291**	0.745**
C18:2c9t11	-0.048	-0.054	0.143*	0.842**	0.760**	-0.103	0.021	0.597**

C20:2n6	0.193*	-0.109	0.155	-0.390**	-0.134	0.612**	-0.558**	0.085
C20:3n6	0.076	0.052	-0.113	0.382**	0.558**	0.010	-0.037	0.497**
C20:3n3	0.187*	-0.041	-0.020	0.109	0.412**	0.858**	-0.140	0.579**
C20:4n6	-0.128	0.348*	-0.233	0.351*	0.351*	-0.338*	0.354*	0.201
C22:2	-0.304**	0.198	-0.086	0.120	0.253*	0.052	-0.181	0.244*
C20:5n3	-0.687**	0.429*	-0.745**	0.430*	0.422*	0.071	0.253	0.391
C22:6n3	0.066	-0.019	0.030	0.079	0.197**	0.206**	-0.056	0.279**
SFA	-0.355**	0.011	0.276**	0.797**	0.701**	-0.354**	0.077	0.416**
MUFA	0.587**	-0.189**	0.309**	0.764**	0.348**	-0.039	0.031	0.278**
PUFA	0.100	0.023	-0.081	0.522**	0.860**	0.504**	-0.143*	0.999**
UFA	0.579**	-0.181**	0.291**	0.793**	0.425**	0.014	0.015	0.371**
MUFA/SFA	0.987**	-0.161**	0.134*	0.239**	0.037	0.155*	-0.018	0.112
PUFA/SFA	0.653**	0.020	-0.217**	-0.430**	-0.225**	0.514**	-0.125*	0.075
UFA/SFA	1.000	-0.139*	0.079	0.133*	-0.009	0.233**	-0.039	0.114
SCFA	-0.139*	1.000	-0.257**	-0.076	0.021	0.009	-0.061	0.022
MCFA	0.079	-0.257**	1.000	0.227**	0.044	-0.222**	0.150*	-0.078
LCFA	0.133*	-0.076	0.227**	1.000	0.725**	-0.190**	0.039	0.522**
n-6	-0.009	0.021	0.044	0.725**	1.000	-0.007	-0.009	0.853**
n-3	0.233**	0.009	-0.222**	-0.190**	-0.007	1.000	-0.260**	0.515**
n-6/n-3	-0.039	-0.061	0.150*	0.039	-0.009	-0.260**	1.000	-0.143*
EFA	0.114	0.022	-0.078	0.522**	0.853**	0.515**	-0.143*	1.000

[1] ** $p < 0.01$. [2] * $p < 0.05$.
