# Supplementary material for: Effect of FABP4 Gene Polymorphisms on Fatty Acid Composition, Chemical Composition, and Carcass Traits in Sonid Sheep
Source: Animals (Basel). 2025 Jan 15;15(2):226. doi: 10.3390/ani15020226 (PMC11758647; doi:10.3390/ani15020226)
Supplement: Supplementary file 1 [file animals-15-00226-s001.zip › Table S5.pdf]

Table S5. Genotypic frequencies, allelic frequencies, and diversity parameters of eight variants in the Sonid population.

Variant	Genotype frequency			Allele frequency		Diversity parameter				
						Ho [1]	He [2]	n_e [3]	PIC [4]	χ^2 [5]
g.57765038C>T	CC	CT	TT	C	T					
	0.015	0.188	0.797	0.109	0.891	0.806	0.194	1.241	0.175	0.244
g.57765008A>G-LD1	AA	AG	GG	A	G					
	0.015	0.181	0.804	0.105	0.895	0.812	0.188	1.232	0.17	0.419
g.57764667T>C	TT	TC	CC	T	C					
	0.904	0.092	0.004	0.95	0.05	0.905	0.095	1.105	0.09	0.177
g.57764632A>G	AA	AG	GG	A	G					
	0.956	0.044	0.000	0.978	0.022	0.957	0.043	1.045	0.042	0.139
g.57764436T>G	TT	TG	GG	T	G					
	0.934	0.066	0.000	0.967	0.033	0.936	0.064	1.069	0.062	0.32
g.57764242G>A	GG	GA	AA	G	A					
	0.328	0.491	0.181	0.574	0.426	0.511	0.489	1.957	0.369	0.003
g.57758026G>A	GG	GA	AA	G	A					
	0.952	0.048	0.000	0.976	0.024	0.953	0.047	1.049	0.046	0.164
g.57757988A>G	AA	AG	GG	A	G					
	0.911	0.085	0.004	0.954	0.046	0.912	0.088	1.096	0.084	0.342

[1] Means that the observed heterozygosity.
[2] Means that the expected heterozygosity.
[3] Means that the effective allele numbers.
[4] Means that the polymorphism information content. The classification was conducted according to the PIC values (PIC value < 0.25, low polymorphism; 0.25 < PIC value < 0.5, moderate polymorphism; and PIC value > 0.5, high polymorphism).
[5] Means that the Hardy-Weinberg equilibrium.
