# Supplementary material for: Effect of FABP4 Gene Polymorphisms on Fatty Acid Composition, Chemical Composition, and Carcass Traits in Sonid Sheep
Source: Animals (Basel). 2025 Jan 15;15(2):226. doi: 10.3390/ani15020226 (PMC11758647; doi:10.3390/ani15020226)
Supplement: Supplementary file 1 [file animals-15-00226-s001.zip › Table S6.pdf]

Table S6. Linkage disequilibrium as measured by D' and r^2 among nine polymorphisms in the *FABP4*.

Variant	g.57757988A>G	g.57758026G>A	g.57764242G>A	g.57764436T>G	g.57764632A>G	g.57764667T>C	g.57764906T>C	g.57765008A>G
g.57758026G>A	D' = 1.000<br>r^2 = 0.001							
g.57764242G>A	D' = 1.000<br>r^2 = 0.035	D' = 1.000<br>r^2 = 0.018						
g.57764436T>G	D' = 1.000<br>r^2 = 0.002	D' = 1.000<br>r^2 = 0.001	D' = 0.827<br>r^2 = 0.031					
g.57764632A>G	D' = 0.172<br>r^2 = 0.000	D' = 0.039<br>r^2 = 0.001	D' = 1.000<br>r^2 = 0.017	D' = 0.020<br>r^2 = 0.000				
g.57764667T>C	D' = 1.000<br>r^2 = 0.922	D' = 1.000<br>r^2 = 0.001	D' = 1.000<br>r^2 = 0.038	D' = 1.000<br>r^2 = 0.002	D' = 0.321<br>r^2 = 0.000			
g.57764906T>C	D' = 1.000<br>r^2 = 0.005	D' = 1.000<br>r^2 = 0.209	D' = 1.000<br>r^2 = 0.086	D' = 1.000<br>r^2 = 0.004	D' = 1.000<br>r^2 = 0.003	D' = 1.000<br>r^2 = 0.006		
g.57765008A>G	D' = 1.000<br>r^2 = 0.005	D' = 1.000<br>r^2 = 0.209	D' = 1.000<br>r^2 = 0.086	D' = 1.000<br>r^2 = 0.004	D' = 1.000<br>r^2 = 0.003	D' = 1.000<br>r^2 = 0.006	D' = 1.000<br>r^2 = 1.000	
g.57765038C>T	D' = 1.000<br>r^2 = 0.006	D' = 1.000<br>r^2 = 0.202	D' = 1.000<br>r^2 = 0.089	D' = 1.000<br>r^2 = 0.004	D' = 1.000<br>r^2 = 0.003	D' = 1.000<br>r^2 = 0.006	D' = 1.000<br>r^2 = 0.962	D' = 1.000<br>r^2 = 0.962
