# Supplementary material for: Effect of FABP4 Gene Polymorphisms on Fatty Acid Composition, Chemical Composition, and Carcass Traits in Sonid Sheep
Source: Animals (Basel). 2025 Jan 15;15(2):226. doi: 10.3390/ani15020226 (PMC11758647; doi:10.3390/ani15020226)
Supplement: Supplementary file 1 [file animals-15-00226-s001.zip › Table S7.pdf]

Table S7. Associations of *FABP4* polymorphisms with FA composition in *longissimus thoracis* muscle in Sonid sheep.

Fatty acid composition (mg/100g)	g.57765038C>T		g.57765008A>G-LD1		g.57764667T>C		g.57764632A>G	
	Genotype		Genotype		Genotype		Genotype	
	CT (51) [1]	TT (216)	AG (49)	GG (218)	TT (245)	TC (25)	AA (259)	AG (12)
C4:0	0.03 ± 0.00	0.03 ± 0.00	0.03 ± 0.00	0.03 ± 0.00	0.03 ± 0.00	0.03 ± 0.00	0.03 ± 0.00	0.03 ± 0.01
C8:0	0.10 ± 0.07	0.23 ± 0.05	0.10 ± 0.07	0.22 ± 0.05	0.20 ± 0.04	0.20 ± 0.14	0.18 ± 0.04 [a]	0.68 ± 0.36 [b]
C10:0	0.19 ± 0.06	0.22 ± 0.03	0.20 ± 0.07	0.22 ± 0.03	0.21 ± 0.03	0.25 ± 0.11	0.21 ± 0.03	0.15 ± 0.08
C12:0	0.04 ± 0.01	0.05 ± 0.01	0.04 ± 0.01	0.05 ± 0.01	0.05 ± 0.01	0.04 ± 0.01	0.05 ± 0.01	0.03 ± 0.01
C14:0	0.39 ± 0.05	0.41 ± 0.02	0.40 ± 0.05	0.41 ± 0.02	0.40 ± 0.02	0.52 ± 0.11	0.41 ± 0.02	0.44 ± 0.06
C15:0	0.15 ± 0.02	0.16 ± 0.01	0.15 ± 0.02	0.16 ± 0.01	0.15 ± 0.01	0.17 ± 0.03	0.16 ± 0.01	0.11 ± 0.03
C16:0	2.03 ± 0.33	2.02 ± 0.14	2.27 ± 0.34	2.21 ± 0.14	2.25 ± 0.14	1.94 ± 0.49	2.20 ± 0.14	2.73 ± 0.45
C17:0	0.20 ± 0.01	0.19 ± 0.00	0.20 ± 0.01	0.19 ± 0.00	0.19 ± 0.00	0.21 ± 0.01	0.19 ± 0.00	0.19 ± 0.01
C18:0	2.03 ± 0.21	2.05 ± 0.08	2.34 ± 0.22	2.45 ± 0.08	2.39 ± 0.07	2.83 ± 0.42	2.44 ± 0.08	2.26 ± 0.28
C22:0	0.05 ± 0.01	0.05 ± 0.00	0.05 ± 0.01	0.05 ± 0.00	0.05 ± 0.00	0.04 ± 0.01	0.05 ± 0.00	0.04 ± 0.01
C23:0	0.10 ± 0.03	0.15 ± 0.02	0.09 ± 0.03 [a]	0.15 ± 0.02 [b]	0.15 ± 0.01	0.08 ± 0.03	0.14 ± 0.01	0.18 ± 0.07
C24:0	0.08 ± 0.00	0.08 ± 0.00	0.08 ± 0.00	0.08 ± 0.00	0.08 ± 0.00	0.09 ± 0.01	0.08 ± 0.00	0.08 ± 0.00
C14:1	0.02 ± 0.00	0.02 ± 0.00	0.02 ± 0.00	0.02 ± 0.00	0.02 ± 0.00 [a]	0.03 ± 0.01 [b]	0.02 ± 0.00	0.01 ± 0.00
C15:1	0.38 ± 0.17	0.14 ± 0.05	0.40 ± 0.18	0.14 ± 0.05	0.17 ± 0.05	0.28 ± 0.27	0.18 ± 0.05	0.20 ± 0.18
C16:1	0.20 ± 0.02a	0.25 ± 0.01b	0.20 ± 0.02 [a]	0.25 ± 0.01 [b]	0.24 ± 0.01	0.25 ± 0.04	0.24 ± 0.01	0.20 ± 0.04
C17:1	0.27 ± 0.08	0.17 ± 0.03	0.28 ± 0.08	0.17 ± 0.03	0.20 ± 0.03	0.11 ± 0.02	0.19 ± 0.03	0.29 ± 0.15
C18:1n9t	1.02 ± 0.29	1.04 ± 0.15	1.20 ± 0.30	1.43 ± 0.15	1.37 ± 0.14	1.61 ± 0.45	1.41 ± 0.14	0.94 ± 0.43
C18:1n9c	0.56 ± 0.21 [a]	1.04 ± 0.18 [b]	0.58 ± 0.22 [a]	1.40 ± 0.18 [b]	1.20 ± 0.16	1.65 ± 0.64	1.24 ± 0.16	1.07 ± 0.65
C18:2n6t	0.02 ± 0.00	0.03 ± 0.00	0.02 ± 0.00	0.03 ± 0.00	0.03 ± 0.00	0.03 ± 0.00	0.03 ± 0.00	0.02 ± 0.00
C18:2n6c	0.76 ± 0.03	0.76 ± 0.02	0.75 ± 0.03	0.76 ± 0.02	0.75 ± 0.01	0.83 ± 0.06	0.75 ± 0.01	0.80 ± 0.06

C18:3n3	0.21 ± 0.01	0.22 ± 0.00	0.21 ± 0.01	0.22 ± 0.00	0.21 ± 0.00	0.24 ± 0.02	0.22 ± 0.00	0.20 ± 0.01
C18:2c9t11	0.17 ± 0.02	0.18 ± 0.01	0.18 ± 0.02	0.18 ± 0.01	0.18 ± 0.01	0.20 ± 0.03	0.18 ± 0.01	0.17 ± 0.02
C20:2n6	0.04 ± 0.01	0.04 ± 0.00	0.04 ± 0.01	0.04 ± 0.00	0.04 ± 0.00	0.05 ± 0.01	0.04 ± 0.00	0.03 ± 0.01
C20:3n3	0.33 ± 0.03	0.28 ± 0.01	0.33 ± 0.03	0.28 ± 0.01	0.28 ± 0.01	0.37 ± 0.04	0.29 ± 0.01	0.24 ± 0.06
C22:6n3	0.01 ± 0.00	0.02 ± 0.00	0.01 ± 0.00	0.02 ± 0.00	0.02 ± 0.00 [A]	0.03 ± 0.00 [B]	0.02 ± 0.00	0.01 ± 0.00
SFA	5.39 ± 0.58	5.64 ± 0.24	5.95 ± 0.60	6.22 ± 0.24	6.15 ± 0.23	6.40 ± 0.89	6.14 ± 0.23	6.92 ± 0.97
MUFA	2.45 ± 0.51	2.67 ± 0.24	2.68 ± 0.52	3.41 ± 0.24	3.20 ± 0.22	3.93 ± 0.84	3.28 ± 0.22	2.71 ± 0.87
PUFA	1.56 ± 0.06	1.55 ± 0.03	1.56 ± 0.06	1.55 ± 0.03	1.53 ± 0.02 [A]	1.76 ± 0.11 [B]	1.55 ± 0.02	1.49 ± 0.10
UFA	4.01 ± 0.53	4.22 ± 0.25	4.24 ± 0.55	4.96 ± 0.25	4.73 ± 0.23	5.69 ± 0.88	4.83 ± 0.23	4.20 ± 0.88
MUFA/SFA	0.45 ± 0.25	0.47 ± 0.06	0.45 ± 0.26	0.55 ± 0.06	0.52 ± 0.07	0.61 ± 0.11	0.53 ± 0.07	0.39 ± 0.44
PUFA/SFA	0.29 ± 0.04 [a]	0.28 ± 0.01 [b]	0.26 ± 0.04	0.25 ± 0.01	0.25 ± 0.01	0.28 ± 0.05	0.25 ± 0.01	0.22 ± 0.08
UFA/SFA	0.74 ± 0.28	0.75± 0.07	0.71 ± 0.29	0.80 ± 0.07	0.77 ± 0.08	0.89 ± 0.13	0.79 ± 0.08	0.61 ± 0.52
SCFA	0.03 ± 0.00	0.03 ± 0.00	0.03 ± 0.00	0.03 ± 0.00	0.03 ± 0.00	0.03 ± 0.00	0.03 ± 0.00	0.03 ± 0.01
MCFA	0.33 ± 0.09	0.50 ± 0.06	0.34 ± 0.09	0.49 ± 0.06	0.46 ± 0.05	0.49 ± 0.17	0.44 ± 0.05	0.86 ± 0.35
LCFA	9.04 ± 0.79	9.32 ± 0.39	9.82 ± 0.82	10.66 ± 0.39	10.39 ± 0.35	11.57 ± 1.50	10.50 ± 0.36	10.23 ± 1.07
n-6	0.84 ± 0.03	0.85 ± 0.02	0.83 ± 0.03	0.85 ± 0.02	0.84 ± 0.02	0.92 ± 0.06	0.84 ± 0.02	0.87 ± 0.06
n-3	0.55 ± 0.03	0.52 ± 0.01	0.55 ± 0.03	0.52 ± 0.01	0.51 ± 0.01 [A]	0.64 ± 0.04 [B]	0.53 ± 0.01	0.45 ± 0.06
n-6/n-3	1.53 ± 0.16	1.63 ± 0.74	1.51 ± 0.16	1.63 ± 0.73	1.65 ± 0.65	1.44 ± 0.20	1.58 ± 0.62	1.93 ± 0.50
EFA	1.39 ± 0.05	1.37 ± 0.02	1.38 ± 0.05	1.37 ± 0.02	1.35 ± 0.02 [A]	1.56 ± 0.09 [B]	1.37 ± 0.02	1.32 ± 0.09

[a, b] Means that the difference between different superscript values within the same line is statistically significant ($p < 0.05$).
[A, B] Means that the difference between different superscript values within the same line is statistically significant ($p < 0.01$).
[1] Represents the mean ± standard error.

Table S7. (Continue)

Faty acid composition (mg/100g)	g.57764436T>G		g.57764242G>A			g.57758026G>A		g.57757988A>G	
	Genotype		Genotype			Genotype		Genotype	
	TT (253) [1]	TG (18)	GG (89)	GA (133)	AA (49)	GG (258)	GA (13)	AA (247)	AG (23)
C4:0	0.03 ± 0.00	0.03 ± 0.01	0.03 ± 0.00	0.03 ± 0.00	0.03 ± 0.00	0.03 ± 0.00	0.03 ± 0.00	0.03 ± 0.00	0.03 ± 0.00
C8:0	0.19 ± 0.04	0.25 ± 0.17	0.30 ± 0.09	0.16 ± 0.06	0.12 ± 0.07	0.19 ± 0.04	0.37 ± 0.26	0.20 ± 0.04	0.22 ± 0.15
C10:0	0.22 ± 0.03	0.04 ± 0.00	0.24 ± 0.06	0.21 ± 0.04	0.17 ± 0.06	0.21 ± 0.03	0.31 ± 0.15	0.21 ± 0.03	0.19 ± 0.09
C12:0	0.05 ± 0.01	0.04 ± 0.01	0.03 ± 0.00	0.06 ± 0.01	0.04 ± 0.01	0.05 ± 0.01	0.03 ± 0.01	0.05 ± 0.01	0.04 ± 0.01
C14:0	0.40 ± 0.02	0.52 ± 0.07	0.46 ± 0.04	0.38 ± 0.02	0.38 ± 0.04	0.40 ± 0.02	0.47 ± 0.09	0.40 ± 0.02	0.54 ± 0.12
C15:0	0.16 ± 0.01 [A]	0.07 ± 0.01 [B]	0.14 ± 0.02	0.16 ± 0.01	0.16 ± 0.02	0.15 ± 0.01	0.18 ± 0.04	0.15 ± 0.01	0.17 ± 0.03
C16:0	2.12 ± 0.14 [A]	3.67 ± 0.35 [B]	2.59 ± 0.25	2.06 ± 0.18	2.02 ± 0.29	2.20 ± 0.13	2.57 ± 0.69	2.24 ± 0.14	2.00 ± 0.53
C17:0	0.19 ± 0.00	0.18 ± 0.02	0.20 ± 0.01	0.19 ± 0.01	0.19 ± 0.01	0.19 ± 0.00	0.19 ± 0.02	0.19 ± 0.00	0.21 ± 0.01
C18:0	2.40 ± 0.08	2.86 ± 0.22	2.67 ± 0.16 [a]	2.31 ± 0.10 [b]	2.33 ± 0.15 [ab]	2.43 ± 0.08	2.47 ± 0.40	2.39 ± 0.07	2.88 ± 0.46
C22:0	0.05 ± 0.00 [A]	0.09 ± 0.01 [B]	0.05 ± 0.00	0.04 ± 0.00	0.05 ± 0.01	0.05 ± 0.00	0.05 ± 0.01	0.05 ± 0.00	0.05 ± 0.01
C23:0	0.12 ± 0.01 [A]	0.39 ± 0.06 [B]	0.15 ± 0.02	0.13 ± 0.02	0.14 ± 0.03	0.14 ± 0.01	0.05 ± 0.04	0.15 ± 0.01	0.09 ± 0.04
C24:0	0.08 ± 0.00	0.07 ± 0.01	0.08 ± 0.00	0.08 ± 0.00	0.08 ± 0.00	0.08 ± 0.00	0.08 ± 0.00	0.08 ± 0.00	0.09 ± 0.01
C14:1	0.02 ± 0.00	0.01 ± 0.00	0.02 ± 0.00	0.02 ± 0.00	0.02 ± 0.00	0.02 ± 0.00	0.03 ± 0.01	0.02 ± 0.00 [a]	0.03 ± 0.01 [b]
C15:1	0.19 ± 0.05	0.02 ± 0.00	0.20 ± 0.10	0.16 ± 0.06	0.19 ± 0.14	0.15 ± 0.05 [A]	0.79 ± 0.42 [B]	0.17 ± 0.05	0.31 ± 0.29
C16:1	0.24 ± 0.01	0.30 ± 0.04	0.25 ± 0.02	0.24 ± 0.01	0.22 ± 0.02	0.24 ± 0.01	0.28 ± 0.05	0.24 ± 0.01	0.25 ± 0.04
C17:1	0.19 ± 0.03	0.17 ± 0.01	0.19 ± 0.04	0.21 ± 0.04	0.14 ± 0.03	0.17 ± 0.02 [A]	0.50 ± 0.27 [B]	0.20 ± 0.03	0.11 ± 0.02
C18:1n9t	1.46 ± 0.14 [a]	0.32 ± 0.04 [b]	1.18 ± 0.21	1.48 ± 0.20	1.48 ± 0.34	1.35 ± 0.13	2.06 ± 0.79	1.38 ± 0.14	1.48 ± 0.45
C18:1n9c	1.20 ± 0.16	1.71 ± 0.61	1.71 ± 0.32 [a]	0.90 ± 0.18 [b]	1.30 ± 0.37 [ab]	1.27 ± 0.16	0.52 ± 0.35	1.19 ± 0.16	1.78 ± 0.69
C18:2n6t	0.03 ± 0.00	0.03 ± 0.00	0.03 ± 0.00	0.03 ± 0.01	0.02 ± 0.00	0.03 ± 0.00	0.03 ± 0.00	0.03 ± 0.00	0.03 ± 0.01
C18:2n6c	0.75 ± 0.01 [A]	0.90 ± 0.07 [B]	0.80 ± 0.03 [a]	0.73 ± 0.02 [b]	0.76 ± 0.03 [ab]	0.76 ± 0.02	0.76 ± 0.04	0.75 ± 0.01	0.84 ± 0.06

C18:3n3	0.21 ± 0.00	0.23 ± 0.01	0.23 ± 0.01 [a]	0.21 ± 0.01 [b]	0.21 ± 0.01 [ab]	0.21 ± 0.00	0.23 ± 0.01	0.21 ± 0.00	0.24 ± 0.02
C18:2c9t11	0.17 ± 0.01	0.22 ± 0.02	0.20 ± 0.01 [a]	0.17 ± 0.01 [b]	0.17 ± 0.02 [ab]	0.18 ± 0.01	0.22 ± 0.04	0.18 ± 0.01	0.20 ± 0.03
C20:2n6	0.04 ± 0.00	0.00 ± 0.00	0.04 ± 0.01	0.04 ± 0.00	0.04 ± 0.01	0.04 ± 0.00	0.05 ± 0.01	0.04 ± 0.00	0.05 ± 0.01
C20:3n3	0.31 ± 0.01 [A]	0.07 ± 0.04 [B]	0.28 ± 0.02	0.29 ± 0.02	0.30 ± 0.03	0.29 ± 0.01	0.34 ± 0.04	0.28 ± 0.01	0.37 ± 0.04
C22:6n3	0.02 ± 0.00	0.02 ± 0.00	0.02 ± 0.00	0.02 ± 0.00	0.02 ± 0.00	0.02 ± 0.00	0.02 ± 0.00	0.02 ± 0.00 [A]	0.03 ± 0.00 [B]
SFA	6.01 ± 0.23 [a]	8.21 ± 0.67 [b]	6.94 ± 0.43 [a]	5.81 ± 0.31 [b]	5.71 ± 0.46 [ab]	6.12 ± 0.23	6.80 ± 1.04	6.14 ± 0.23	6.51 ± 0.96
MUFA	3.30 ± 0.23	2.53 ± 0.61	3.55 ± 0.40	3.01 ± 0.29	3.35 ± 0.53	3.20 ± 0.22	4.18 ± 1.26	3.20 ± 0.22	3.96 ± 0.90
PUFA	1.55 ± 0.02	1.50 ± 0.10	1.62 ± 0.05 [a]	1.51 ± 0.03 [b]	1.54 ± 0.05 [ab]	1.55 ± 0.02	1.66 ± 0.12	1.53 ± 0.02 [A]	1.77 ± 0.12 [B]
UFA	4.85 ± 0.23	4.03 ± 0.61	5.17 ± 0.42	4.52 ± 0.30	4.89 ± 0.55	4.75 ± 0.23	5.84 ± 1.31	4.73 ± 0.23	5.73 ± 0.94
MUFA/SFA	0.55 ± 0.07	0.31 ± 0.07	0.51 ± 0.13	0.52 ± 0.10	0.59 ± 0.11	0.52 ± 0.06 [A]	0.61 ± 0.82 [B]	0.52 ± 0.07	0.61 ± 0.12
PUFA/SFA	0.26 ± 0.01 [A]	0.18 ± 0.01 [B]	0.23 ± 0.03	0.26 ± 0.02	0.27 ± 0.03	0.25 ± 0.01	0.24 ± 0.12	0.25 ± 0.01	0.27 ± 0.05
UFA/SFA	0.81 ± 0.08	0.49 ± 0.07	0.74 ± 0.15	0.78 ± 0.11	0.86 ± 0.12	0.78 ± 0.07 [A]	0.86 ± 0.93 [B]	0.77 ± 0.08	0.88 ± 0.14
SCFA	0.03 ± 0.00	0.03 ± 0.01	0.03 ± 0.00	0.03 ± 0.00	0.03 ± 0.00	0.03 ± 0.00	0.03 ± 0.00	0.03 ± 0.00	0.03 ± 0.00
MCFA	0.46 ± 0.05	0.33 ± 0.17	0.57 ± 0.10	0.43 ± 0.07	0.33 ± 0.09	0.45 ± 0.05	0.71 ± 0.26	0.46 ± 0.05	0.45 ± 0.17
LCFA	10.37 ± 0.36	11.88 ± 1.04	11.51 ± 0.68 [a]	9.87 ± 0.44 [b]	10.24 ± 0.83 [ab]	10.39 ± 0.36	11.90 ± 1.18	10.38 ± 0.35	11.76 ± 1.62
n-6	0.84 ± 0.01 [a]	0.96 ± 0.08 [b]	0.89 ± 0.03 [a]	0.82 ± 0.02 [b]	0.84 ± 0.03 [ab]	0.85 ± 0.02	0.85 ± 0.05	0.84 ± 0.02	0.93 ± 0.07
n-3	0.54 ± 0.01 [A]	0.32 ± 0.04 [B]	0.53 ± 0.02	0.52 ± 0.02	0.53 ± 0.03	0.52 ± 0.01	0.59 ± 0.06	0.51 ± 0.01 [A]	0.64 ± 0.05 [B]
n-6/n-3	1.56 ± 0.63	3.00 ± 0.36	1.68 ± 0.13	1.58 ± 1.18	1.58 ± 0.17	1.63 ± 0.62	1.44 ± 0.30	1.65 ± 0.65	1.45 ± 0.21
EFA	1.38 ± 0.02	1.28 ± 0.09	1.42 ± 0.04	1.34 ± 0.03	1.38 ± 0.04	1.37 ± 0.02	1.44 ± 0.09	1.35 ± 0.02 [A]	1.57 ± 0.10 [B]

[a, b] Means that the difference between different superscript values within the same line is statistically significant ($p < 0.05$).
[A, B] Means that the difference between different superscript values within the same line is statistically significant ($p < 0.01$).
[1] Represents the mean ± standard error.
