# Supplementary material for: Effect of FABP4 Gene Polymorphisms on Fatty Acid Composition, Chemical Composition, and Carcass Traits in Sonid Sheep
Source: Animals (Basel). 2025 Jan 15;15(2):226. doi: 10.3390/ani15020226 (PMC11758647; doi:10.3390/ani15020226)
Supplement: Supplementary file 1 [file animals-15-00226-s001.zip › Table S8.pdf]

Table S8. Association of *FABP4* polymorphisms with chemical composition in *longissimus thoracis* muscle of Sonid sheep.

Chemical composition		g.57765038C>T		g.57765008A>G-LD1		g.57764667T>C		g.57764436T>G	
		Genotype		Genotype		Genotype		Genotype	
		CT (18) [1]	TT (91)	AG (18)	GG (91)	TT (95)	TC (16)	TT (92)	TG (19)
Crude protein (%)		21.12 ± 0.33	21.11 ± 0.13	21.12 ± 0.33	21.11 ± 0.13	21.13 ± 0.13	21.04 ± 0.51	21.16 ± 0.13	20.66 ± 0.33
Crude fat (g/100g)		6.80 ± 0.34	6.97 ± 0.12	6.84 ± 0.35	6.96 ± 0.12	6.90 ± 0.12	7.79 ± 0.50	6.96 ± 0.12	6.94 ± 0.31
Free amino acid (ng/0.1g)	Asp	63.49 ± 1.13	46.62 ± 5.62	46.34 ± 5.97	55.10 ± 6.95	53.47 ± 6.13	59.72 ± 4.41	49.66 ± 2.67 [a]	95.74 ± 58.72 [b]
	Thr	54.62 ± 6.67	54.17 ± 2.36	54.52 ± 7.07	54.19 ± 2.33	54.69 ± 2.37	47.09 ± 5.24	53.29 ± 2.31	67.34 ± 8.50
	Ser	123.72 ± 7.88	144.73 ± 7.43	123.43 ± 8.35	144.53 ± 7.35	140.46 ± 6.07	144.13 ± 42.82	140.00 ± 6.35	147.61 ± 25.20
	Glu	388.48 ± 39.42	342.44 ± 17.84	389.99 ± 41.79	342.69 ± 17.63	357.84 ± 16.93	308.80 ± 58.83	358.92 ± 16.95	314.17 ± 59.71
	Gly	303.76 ± 19.94	319.71 ± 7.90	298.50 ± 20.40	320.61 ± 7.86	318.36 ± 7.60	296.84 ± 20.30	313.15 ± 7.17	357.27 ± 34.27
	Ala	2019.27 ± 84.72	2059.50 ± 46.19	2020.21 ± 89.86	2058.81 ± 45.63	2061.85 ± 42.08	1974.30 ± 105.74	2042.17 ± 35.20	2204.71 ± 279.04
	Cys	20.43 ± 2.09	19.64 ± 0.60	20.37 ± 2.21	19.66 ± 0.59	19.92 ± 0.64	19.01 ± 1.40	19.65 ± 0.62	22.08 ± 2.41
	Val	394.89 ± 13.25	396.44 ± 8.11	393.16 ± 13.93	396.78 ± 8.01	397.46 ± 7.15	379.66 ± 26.45	396.95 ± 6.17	390.81 ± 47.21
	Met	137.24 ± 10.26	134.66 ± 3.77	135.60 ± 10.74	135.03 ± 3.75	135.56 ± 3.70	132.95 ± 10.06	134.84 ± 3.37	141.16 ± 20.35
	Ile	275.65 ± 13.22	274.11 ± 5.36	273.07 ± 13.75	274.67 ± 5.32	275.90 ± 5.13	260.11 ± 14.04	272.87 ± 4.86	298.94 ± 24.89
	Leu	699.59 ± 41.54	679.82 ± 16.19	692.01 ± 43.31	681.64 ± 16.10	687.09 ± 15.77	661.43 ± 37.41	684.46 ± 13.37	696.90 ± 103.36
	Tyr	143.41 ± 6.53	140.05 ± 3.67	143.00 ± 6.92	140.18 ± 3.62	142.31 ± 3.35	128.79 ± 9.71	141.15 ± 3.01	145.16 ± 19.81

Phe	203.10 ± 16.95	192.48 ± 6.00	202.11 ± 17.95	192.82 ± 5.94	196.19 ± 6.06	187.35 ± 19.87	196.01 ± 5.81	192.12 ± 28.42
Lys	1284.60 ± 51.56	1258.78 ± 33.52	1287.65 ± 54.59	1258.46 ± 33.10	1258.07 ± 29.04	1336.40 ± 133.73	1245.22 ± 28.23 [a]	1464.63 ± 128.07 [b]
NH3	1533.82 ± 50.08	1510.19 ± 25.41	1525.99 ± 52.47	1512.11 ± 25.17	1519.12 ± 22.84	1421.23 ± 93.20	1513.98 ± 21.73	1506.42 ± 118.90
His	68.52 ± 6.41	70.60 ± 2.42	67.93 ± 6.77	70.69 ± 2.40	70.85 ± 2.41	71.01 ± 5.88	70.80 ± 2.42	71.44 ± 7.39
Arg	421.64 ± 23.32	424.91 ± 10.53	423.03 ± 24.69	424.59 ± 10.41	427.09 ± 9.82	385.63 ± 23.20	426.34 ± 8.69	407.11 ± 59.02
Pro	46.01 ± 6.84	53.53 ± 4.33	45.03 ± 7.18	53.65 ± 4.28	52.09 ± 3.76	47.74 ± 19.13	51.69 ± 3.72	53.42 ± 18.32

[a, b] Means that the difference between different superscript values within the same line is statistically significant ($p < 0.05$).
[1] Represents the mean ± standard error.

Table S8. (Continue)

Chemical composition		g.57764242G>A			g.57758026G>A		g.57757988A>G	
		Genotype			Genotype		Genotype	
		GG (18)	GA (55)	AA (38)	GG (96)	GA (15)	AA (95)	AG (16)
Crude protein (%)		20.88 ± 0.22 [a]	21.13 ± 0.17 [ab]	21.60 ± 0.27 [b]	21.15 ± 0.12	20.27 ± 0.57	21.13 ± 0.13	21.04 ± 0.51
Crude fat (g/100g)		7.43 ± 0.21 [a]	6.75 ± 0.15 [b]	6.59 ± 0.27 [b]	6.93 ± 0.12	7.75 ± 0.28	6.90 ± 0.12	7.79 ± 0.50
Free amino acid (ng/0.1g)	Asp	83.34 ± 34.68 [a]	47.08 ± 3.71 [b]	50.90 ± 4.27 [ab]	54.33 ± 5.98	42.39 ± 15.99	53.47 ± 6.13	59.72 ± 4.41
	Thr	60.91 ± 6.24	52.77 ± 3.02	53.84 ± 3.98	54.37 ± 2.13	51.18 ± 20.48	54.69 ± 2.37	47.09 ± 5.24
	Ser	135.85 ± 15.63	144.82 ± 9.65	136.47 ± 8.64	140.35 ± 6.39	146.97 ± 24.36	140.46 ± 6.07	144.13 ± 42.82
	Glu	338.83 ± 41.46	363.78 ± 23.17	348.49 ± 28.06	348.42 ± 16.73	479.86 ± 47.05	357.84 ± 16.93	308.80 ± 58.83
	Gly	326.76 ± 22.10	319.31 ± 10.73	309.41 ± 10.32	320.20 ± 7.31	257.29 ± 35.70	318.36 ± 7.60	296.84 ± 20.30
	Ala	2118.71 ± 162.87	2027.49 ± 48.53	2073.87 ± 61.66	2075.41 ± 40.78 [a]	1696.45 ± 131.76 [b]	2061.85 ± 42.08	1974.30 ± 105.74
	Cys	21.72 ± 2.14	19.64 ± 0.83	19.40 ± 0.88	19.95 ± 0.62	18.31 ± 2.95	19.92 ± 0.64	19.01 ± 1.40
	Val	407.51 ± 29.76	395.47 ± 8.33	392.92 ± 9.79	395.33 ± 7.15	416.89 ± 23.95	397.46 ± 7.15	379.66 ± 26.45
	Met	140.89 ± 11.45	136.08 ± 4.21	131.94 ± 6.65	134.65 ± 3.52	149.77 ± 23.32	135.56 ± 3.70	132.95 ± 10.06
	Ile	290.41 ± 15.24	273.23 ± 6.75	271.22 ± 7.92	274.55 ± 5.01	282.70 ± 25.34	275.90 ± 5.13	260.11 ± 14.04
	Leu	684.83 ± 60.85	690.49 ± 17.94	678.37 ± 23.74	683.66 ± 15.22	722.21 ± 86.06	687.09 ± 15.77	661.43 ± 37.41
	Tyr	151.39 ± 11.40	139.49 ± 3.87	140.24 ± 5.61	141.65 ± 3.37	138.74 ± 5.82	142.31 ± 3.35	128.79 ± 9.71

Phe	200.61 ± 16.57	197.07 ± 7.65	191.33 ± 10.62	194.16 ± 5.79	224.47 ± 38.84	196.19 ± 6.06	187.35 ± 19.87
Lys	1348.55 ± 85.35	1235.97 ± 45.24	1268.45 ± 30.58	1255.31 ± 28.99	1404.62 ± 129.27	1258.07 ± 29.04	1336.40 ± 133.73
NH3	1509.68 ± 77.50	1494.51 ± 28.28	1543.66 ± 37.04	1519.71 ± 22.13	1390.41 ± 145.90	1519.12 ± 22.84	1421.23 ± 93.20
His	68.52 ± 5.47	69.18 ± 2.89	74.37 ± 4.58	70.61 ± 2.28	75.51 ± 16.31	70.85 ± 2.41	71.01 ± 5.88
Arg	425.42 ± 34.57	425.06 ± 10.04	423.60 ± 18.11	422.22 ± 9.42	470.89 ± 57.04	427.09 ± 9.82	385.63 ± 23.20
Pro	55.32 ± 10.95	49.12 ± 5.16	54.53 ± 6.07	53.39 ± 3.80	22.14 ± 6.49	52.09 ± 3.76	47.74 ± 19.13

[a, b] Means that the difference between different superscript values within the same line is statistically significant ($p < 0.05$).
