# Supplementary material for: Effect of FABP4 Gene Polymorphisms on Fatty Acid Composition, Chemical Composition, and Carcass Traits in Sonid Sheep
Source: Animals (Basel). 2025 Jan 15;15(2):226. doi: 10.3390/ani15020226 (PMC11758647; doi:10.3390/ani15020226)
Supplement: Supplementary file 1 [file animals-15-00226-s001.zip › Table S9.pdf]

Table S9. Association of *FABP4* polymorphisms with carcass traits in Sonid sheep.

**Carcass trait** [1]	**g.57765038C>T**		**g.57765008A>G-LD1**		**g.57764667T>C**	
	Genotype		Genotype		Genotype	
	CT (18) [2]	TT (91)	AG (18)	GG (91)	TT (95)	TC (16)
*L** (lightness)	23.86 ± 0.51	23.01 ± 0.33	23.86 ± 0.51	23.01 ± 0.33	23.01 ± 0.31	24.28 ± 0.73
*a** (redness)	13.76 ± 0.46	13.97 ± 0.29	13.76 ± 0.46	13.97 ± 0.29	13.98 ± 0.27	13.80 ± 0.67
*b** (yellowness)	4.68 ± 0.40	5.10 ± 0.14	4.68 ± 0.40	5.10 ± 0.14	5.07 ± 0.14	4.93 ± 0.35
pH_1 (at 45 min)	6.52 ± 0.07	6.56 ± 0.03	6.51 ± 0.08	6.56 ± 0.03	6.56 ± 0.03	6.56 ± 0.09
pH_2 (at 24 h)	5.39 ± 0.02	5.42 ± 0.01	5.38 ± 0.02	5.42 ± 0.01	5.41 ± 0.01	5.46 ± 0.05
Carcass weight (kg)	11.31 ± 0.55	10.76 ± 0.27	11.43 ± 0.59	10.74 ± 0.27	11.10 ± 0.24 ^{a}	9.33 ± 0.80 ^{b}
Live weight (kg)	26.75 ± 1.09	24.68 ± 0.50	27.03 ± 1.15	24.66 ± 0.49	25.67 ± 0.45 ^{A}	21.14 ± 1.50 ^{B}
Dressing percentage	0.42 ± 0.01	0.44 ± 0.01	0.42 ± 0.01	0.44 ± 0.01	0.43 ± 0.01	0.44 ± 0.03

$^{a, b}$ Means that the difference between different superscript values within the same line is statistically significant ($p < 0.05$).
$^{A, B}$ Means that the difference between different superscript values within the same line is statistically significant ($p < 0.01$).
[1] The *L**, *a**, *b**, pH_1, and pH_2 were determined on the *longissimus thoracis* muscle in Sonid sheep.
[2] Represents the mean ± standard error.

Table S9. (Continue)

**Carcass trait** [1]	**g.57764242G>A**			**g.57758026G>A**		**g.57757988A>G**	
	Genotype			Genotype		Genotype	
	GG (18)[2]	GA (55)	AA (38)	GG (96)	GA (15)	AA (95)	AG (16)
*L** (lightness)	23.22 ± 0.49	23.12 ± 0.39	23.13 ± 0.85	23.09 ± 0.30	24.76 ± 0.51	23.01 ± 0.31	24.28 ± 0.73
*a** (redness)	13.75 ± 0.63	14.03 ± 0.27	14.13 ± 0.48	13.99 ± 0.26	13.05 ± 0.98	13.98 ± 0.27	13.80 ± 0.67
*b** (yellowness)	5.20 ± 0.22	5.10 ± 0.19	4.69 ± 0.24	5.09 ± 0.13	4.04 ± 0.54	5.07 ± 0.14	4.93 ± 0.35
pH_1 (at 45 min)	6.55 ± 0.05	6.53 ± 0.03	6.66 ± 0.06	6.56 ± 0.02	6.30 ± 0.24	6.56 ± 0.03	6.56 ± 0.09
pH_2 (at 24 h)	5.41 ± 0.01	5.43 ± 0.02	5.39 ± 0.02	5.42 ± 0.01	5.38 ± 0.03	5.41 ± 0.01	5.46 ± 0.05
Carcass weight (kg)	11.84 ± 0.58 [a]	10.87 ± 0.31 [ab]	9.89 ± 0.41 [b]	10.88 ± 0.25	12.00 ± 0.00	11.10 ± 0.24 [a]	9.33 ± 0.80 [b]
Live weight (kg)	26.53 ± 0.93 [a]	25.15 ± 0.61 [ab]	23.56 ± 0.97 [b]	25.13 ± 0.47	26.65 ± 0.00	25.67 ± 0.45 [A]	21.14 ± 1.50 [B]
Dressing percentage	0.45 ± 0.02	0.43 ± 0.01	0.42 ± 0.01	0.43 ± 0.01	0.45 ± 0.00	0.43 ± 0.01	0.44 ± 0.03

[a, b] Means that the difference between different superscript values within the same line is statistically significant ($p < 0.05$).
[A, B] Means that the difference between different superscript values within the same line is statistically significant ($p < 0.01$).
[1] The *L**, *a**, *b**, pH_1, and pH_2 were determined on the *longissimus thoracis* muscle in Sonid sheep.
[2] Represents the mean ± standard error.
